# Glial *Bmal1* role in mammalian retina daily changes

**DOI:** 10.1038/s41598-022-25783-1

**Published:** 2022-12-13

**Authors:** Serena Riccitelli, Fabio Boi, Davide Lonardoni, Lidia Giantomasi, Olga Barca-Mayo, Davide De Pietri Tonelli, Silvia Bisti, Stefano Di Marco, Luca Berdondini

**Affiliations:** 1 NetS3 Lab, Fondazione Istituto Italiano di Tecnologia, Genova, Italy; 2 Department of Biotechnology and Applied Clinical Sciences, University of L’Aquila, L’Aquila, Italy; 3 Neurobiology of miRNA Lab, Fondazione Istituto Italiano di Tecnologia, Genova, Italy; 4 Istituto Nazionale di Biostrutture e Biosistemi (INBB), Roma, Italy; 5 Center for Synaptic Neuroscience and Technology, Fondazione Istituto Italiano di Tecnologia, Genova, Italy; 6 Circadian and Glial Biology Lab, University of Santiago de Compostela, Santiago de Compostela, Spain

**Keywords:** Astrocyte, Circadian rhythms and sleep, Retina

## Abstract

Visual information processing in the retina requires the rhythmic expression of clock genes. The intrinsic retinal circadian clock is independent of the master clock located in the hypothalamic suprachiasmatic nucleus and emerges from retinal cells, including glia. Less clear is how glial oscillators influence the daily regulation of visual information processing in the mouse retina. Here, we demonstrate that the adult conditional deletion of the gene *Bmal1* in GLAST-positive glial cells alters retinal physiology. Specifically, such deletion was sufficient to lower the amplitude of the electroretinogram b-wave recorded under light-adapted conditions. Furthermore, recordings from > 20,000 retinal ganglion cells (RGCs), the retina output, showed a non-uniform effect on RGCs activity in response to light across different cell types and over a 24-h period. Overall, our results suggest a new role of a glial circadian gene in adjusting mammalian retinal output throughout the night-day cycle.

## Introduction

All living organisms are exposed to daily environmental changes^1^ and set the time at which physiological and behavioral events occur through internal self-regulating timekeeping mechanisms with respect to the 24 h period. In mammals, the central organizer of the timekeeping system is in the suprachiasmatic nucleus (SCN) located in the hypothalamus^2^, while independent oscillatory systems are located in many peripheral organs and tissues^3^. The synchronization between environmental cycles and endogenous oscillators in the SCN is regulated by different factors, e.g., sleep deprivation, social interactions, behavioral activity, aging, and food availability^4^. Among them, the external day-night cycle is considered the most relevant^5,6^. A cycle of the internal timekeeping mechanism completes in ~ 24 h and, at the molecular level, it arises from self-sustained intracellular transcription-translation feedback loops that involve the essential clock transcription factors CLOCK (Circadian Locomotor Output Cycles Kaput) and BMAL1 (Brain and Muscle Aryl hydrocarbon receptor nuclear translocator-Like protein 1, also known as ARNTL1). They heterodimerize and constitute the positive loop of the molecular clock, enhancing the transcription of target genes, including *Per* and *Cry*. Negative feedback is achieved by PER: CRY heterodimers translocated back to the nucleus to inhibit their transcription. Rhythmic expression of clock genes drives the transcription of thousands of clock-controlled genes (CCGs) that are not directly involved in modulating the circadian rhythm itself but generate rhythms in physiological processes in the brain and peripheral tissues, including the retina^7,8^.

The mammalian retina has an autonomous circadian clock^7,9,10^ that escapes the control of the hypothalamic SCN, and light has been suggested to control retinal circadian rhythmicity directly^11^. Although fundamental retinal processes—from disc shedding to the release of melatonin and dopamine^12^—are under intrinsic self-sustained circadian control, the contribution of different retinal cell clocks to the diurnal control of visual information processing remains unclear. Daily modulation of retinal physiology is manifested as circadian variation in the amplitude of some components of the in vivo electroretinogram (ERG)^13^, in the circadian tuning of photopic and mesopic retinal responses^14^, as well as by an altered retinal function observed upon the constitutive knockdown of *Bmal1* gene in the retina^11^.

Recent publications have shown that glial cells are part of the brain circadian circuit regulating daily behaviors^15–18^. In particular, we have recently provided evidence that the conditional deletion of *Bmal1* in Glutamate Aspartate Transporter positive (GLAST^+^) astrocytes modifies circadian locomotor activity and cognitive function in adult mice^17^. However, despite the well-known importance of glial-neuron communication in the mammalian retina^19,20^, glial contribution to the regulation of day-night retinal rhythms remains unknown.

In the retina, GLAST-positive glial cells include astrocytes and Müller cells^21^. Astrocytes are confined to the nerve fiber layer (NFL) at the inner border of the retina, mainly contacting axons of retinal ganglion cells (RGCs)^22,23^. In contrast, a unique population of glia, the Müller cells, resides in a strategic position spanning the entire retina from the outer to the inner limiting membrane and playing a pivotal role in maintaining retinal function and homeostasis in health and disease^24,25^. Interestingly, isolated Müller cells exhibit circadian rhythms in clock gene expression, and *Bmal1* is necessary for robust circadian rhythms in mouse and human Müller cells^26^.

Here, we report that the conditional deletion of the gene *Bmal1* in astrocytes and Müller cells of adult mice retina is sufficient to alter the organization of retinal clock genes expression pattern throughout the day and retinal function, as revealed by electrophysiological recordings obtained with in vivo ERG and ex vivo high-density multielectrode arrays (HD-MEA).

## Results

### Selective deletion of *Bmal1* in retinal astrocytes and Müller cells decreases the b-wave amplitude under light-adapted conditions

**Figure 1:**
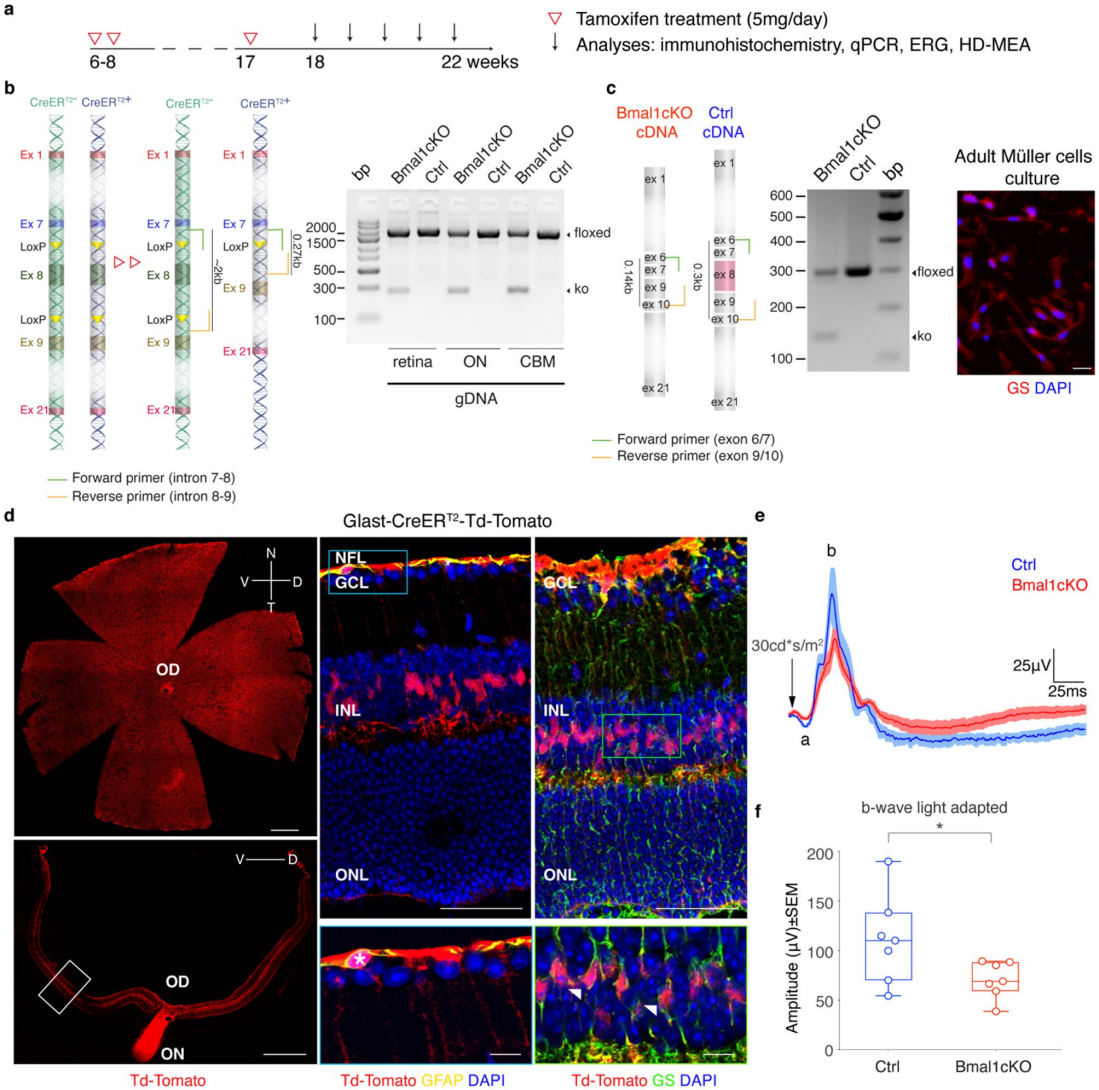
*Bmal1* exon 8 deletion in GLAST^+^ retina cells (astrocytes and Müller cells) reduces b-wave amplitude in photopic ERG. **(a)** Schematic representation of the experimental design. Ctrl and *Bmal1*cKO mice were orally administered on postnatal weeks 6–8 with tamoxifen (5 mg) for 2 consecutive days. A third treatment was repeated one week before the experiments. **(b)** DNA schematics (left panel): transparent color boxes, introns; solid color boxes, exons; triangles, lox P sites; black bars, sizes of PCR products diagnostic of genotypes. PCR products amplified from the retina, optic nerve (ON), and cerebellum (CBM) genomic DNA (gDNA) demonstrate the deletion of exon 8 in *Bmal1*cKO mice (0.27 kb) after TM treatment. **(c)** cDNA schematics: grey and red boxes, exons; black bars, sizes of PCR products. *Bmal1* exon 8 deletion from adult Müller cells of *Bmal1*cKO after TM treatment was tested by PCR using primers described in the figure (left). Amplified PCR products show fragments diagnostic of disrupted Bmal1 allele in *Bmal1*cKO (PCR product 0.14 kb). On the right, representative immunocytochemistry indicates cultured cells as Müller cells GS positive (red). Scale bar, 20 μm. **(d)** Glast-CreER^T2^-Td-Tomato reporter expression throughout the retina, in the whole mount, and the transverse retina (left panel) following TM treatment. Cre recombinase activity is confined explicitly to astrocytes GFAP positive (yellow, a white asterisk in the magnification below) and Müller Cells GS positive (green, white arrow in the magnification below) (right panels). GFAP, Glial fibrillary acidic protein; GS, Glutamine synthetase; OD and ON, optic disc and nerve; GCL, ganglion cell layer; INL, inner nuclear layer, and ONL, outer nuclear layer. Scale bars: left 500 μm, right upper 50 μm, and right bottom 10 μm. **(e)** Photopic light-adapted ERG averaged waveforms (mean ± SEM) elicited by 1 Hz flashes of 30 cd s/m^2^ for Ctrl (n = 7, blue) and *Bmal1*cKO (n = 7, red) mice (a-and b-waves are labeled on the waveforms). **(f)** Quantification of fERG responses showing b-wave amplitude reduction by ~ 36% in *Bmal1*cKO mice under light-adapted conditions (cone pathway ERG). Data are shown as mean ± SEM; animals were recorded at ZT6, *P < 0.05 vs. Ctrl, Student’s *t*-test.

To evaluate the glial contribution to the day-night retinal changes, we conditionally deleted the core clock gene *Bmal1* in astrocytes and Müller cells by crossing a knock-in mouse line bearing a Tamoxifen (TM)-inducible CreER^T2^ recombinase under the control of *Glutamate Aspartate Transporter* (*Glast)* promoter^27^, with a mouse carrying a floxed basic helix-loop-helix *Bmal1* domain allele (corresponding to the exon 8)^11^ as we previously reported^17,28^. Two months after oral TM treatment to 6 to 8-weeks-old mice, the deletion of the exon 8 of the *Bmal1* gene from GLAST^+^ cells was ascertained at the DNA level (Fig. 1a,b, DNA schematics in the left panel). Analysis of genomic DNA performed on retinae, optic nerves (ON), and cerebellum (CBM) of TM-treated *Glast*-CreER^T2^; *Bmal1*^*f*lx/*flx*^ (here referred to as *Bmal1*cKO) and TM-treated *Bmal1*^*flx/flx*^ mice (here referred to as Ctrl) showed that GLAST positive tissues^29^ present an amplified band in correspondence of the 0.27 kb only in *Bmal1*cKO, indicating the excision of the floxed allele, together with a ~ 2 kb indicative of GLAST negative cells that did not undergo TM-induced Cre-mediated recombination (Fig. 1b, right panel and Supplementary Fig. S2a). To further test the specificity of the deletion, Müller glial cells were isolated from retinal explants of adult *Bmal1*cKO and Ctrl mice after TM treatment and cultured. PCR products from cultured cells cDNA (obtained after RNA extraction) showed *Bmal1* exon 8 excision (Fig. 1c, cDNA schematics and in Fig. 1c, central panel a size band of 0.14 kb and Supplementary Fig. S2b) only in *Bmal1*cKO cDNA. A 0.3 kb band, indicating the presence of the exon 8, was detected even in conditional *Bmal1*cKO samples suggesting either that TM did not elicit Cre-mediated recombination in every GLAST-expressing cell or contamination of GLAST negative cells in the adult primary culture.

Furthermore, the specificity of Cre expression in the mouse retina was examined by immunohistochemical analysis studies of retinae from *Glast*-CreER^T2^ mice crossed with a Cre-inducible red fluorescent reporter mouse line (Td-Tomato)^17^. Efficient expression of Cre-recombined cells was observed 2 months after TM treatment, as revealed by the Td-Tomato signal (red) that spans throughout the whole-mount retina (Fig. 1d, left upper panel). Analysis of vertical sections across the retinae (Fig. 1d, right panel) showed that Td-Tomato-positive cells specifically colocalize with the astrocytic marker GFAP (Glial fibrillary acidic protein, in yellow) confined in the nerve fiber layer (NFL) and a Müller cells marker (GS, Glutamine Synthetase, in green) which somata are located in the inner nuclear layer (INL), while the processes span through the entire retina intimately connecting photoreceptors and other neurons^25,30^. No other cells, except astrocytes and Müller cells, were Td-Tomato positive. Together with the above-presented evidence, this approach effectively induces the selective deletion of *Bmal1* in GLAST^+^ astrocytes and Müller cells of adult mice retinae upon TM treatment.

To examine the involvement of glial *Bmal1* in the modulation of the retinal response to light, we carried out in vivo ERG recordings in *Bmal1*cKO and compared them with littermate controls. Recordings were performed in “day-dark” conditions as Barnard et al.^13^, with mice maintained in darkness overnight and recorded during mid-day hours, after 10 min of light adaptation as this provides the maximal photopic b-wave amplitude (photopic ERG)^11,13^. Results showed a reduced b-wave amplitude by ~ 36% (Fig. 1e,f n = 7/7 Ctrl and *Bmal1*cKO, mean ± SEM 111.1 ± 16.90 vs. 70.91 ± 6.97, respectively; P = 0.0483, Student’s *t*-test) at 30 cd s/m^2^ in *Bmal1*cKO compared to Ctrl mice, while no significant differences were found analyzing both the a- and b-wave amplitudes in scotopic condition (0.001 to 30 cd s/m^2^, Supplementary Fig. S1).

### Altered response to light is not driven by significant changes in retinal morphology or activation of inflammatory processes

**Figure 2:**
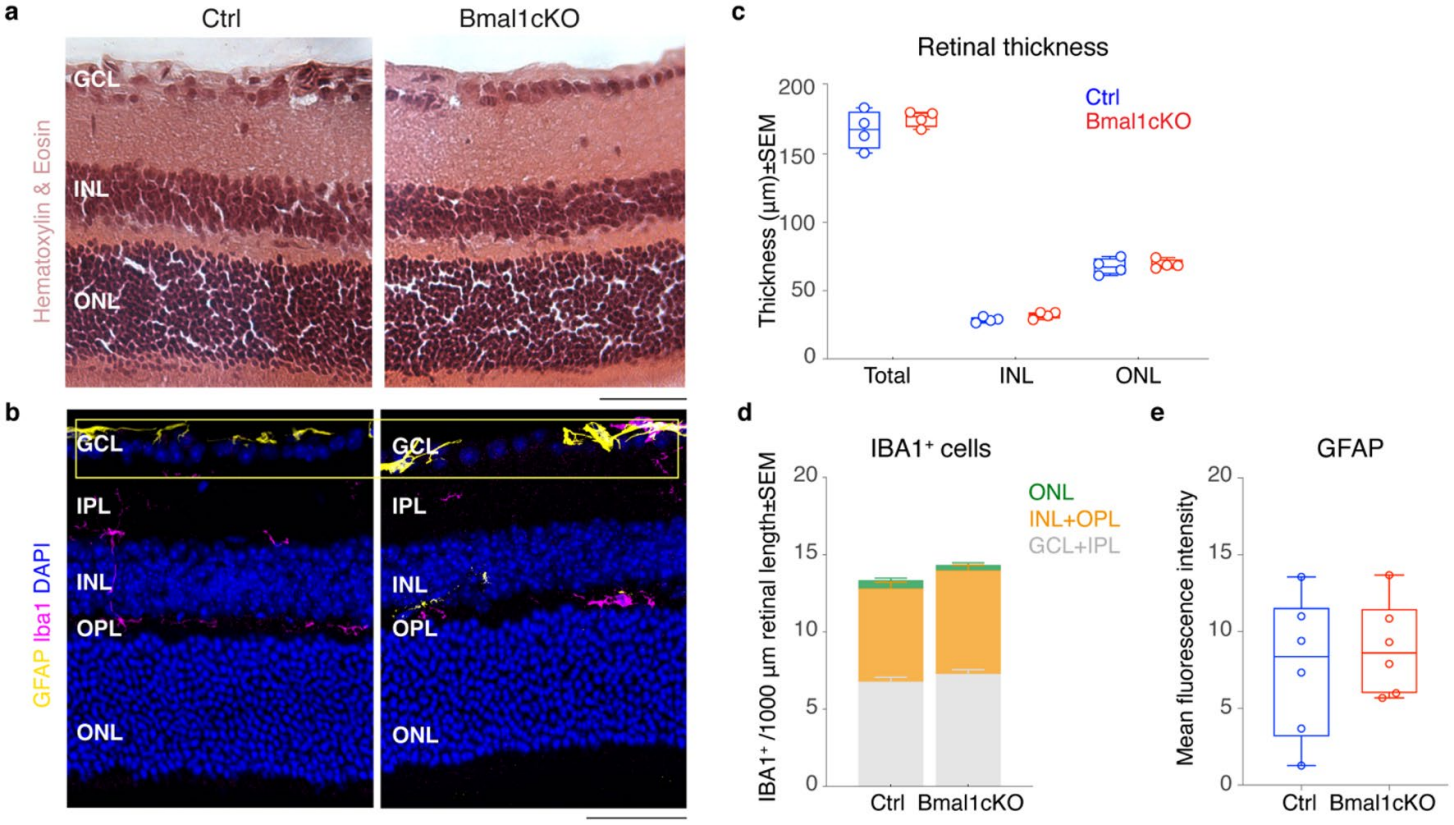
GLAST^+^ cells specific deletion of Bmal1 does not induce changes in retinal morphological or glial cell reactivity. **(a)** Representative transverse retinal sections from Ctrl and *Bmal1*cKO stained with Hematoxylin&Eosin to show the cellular and extracellular morphology. **(b)** Fluorescence images of retinal sections stained with DAPI (blue) to reveal cell nuclei. Ctrl and *Bmal1*cKO retinal sections do not show any apparent difference in neural architecture. IBA1^+^ cells (pink) are localized in the INL, GCL, and plexiform layers. Any noticeable structural changes in the microglial cells are detectable. GFAP immunoreactivity (yellow) is confined in the astrocytic layer in both conditions (yellow box), indicating the innermost retinal layer astrocyte population. Not detected reactivity of Müller cells. **(c)** Quantitative comparison of the total retina, INL, and ONL thickness. No statistically significant differences were detected between genotypes. n = 4/4, P > 0.05 vs. Ctrl, Student’s *t*-test. **(d)** Quantitative analysis of the number of IBA1^+^ cells in each layer per 1000 μm segment of retina. No statistically significant differences were detected between genotypes. n = 8/8, P > 0.05 vs. Ctrl, Student’s *t*-test. **(e)** Graphs showing GFAP immunoreactivity in Ctrl and *Bmal1*cKO per 15e + 3 μm^2^, including the inner and outer retina. No statistically significant differences were detected between genotypes. n = 6/6, P > 0.05 vs. Ctrl, Student’s *t*-test. *ONL* outer nuclear layer, *INL* inner nuclear layer, *GCL* ganglion cell layer, *OPL* outer plexiform layer, *IPL* inner plexiform layer, *GFAP* glial fibrillary acidic protein, *IBA1* ionized calcium-binding adapter molecule 1. Data are shown as mean ± SEM. Scale bar, 50 μm.

To examine the cellular basis of the reduced photopic ERGs b-wave amplitude in *Bmal1*cKO, we assessed retina morphology, astrocytes reactivity^28,31^ and microglia activation (assessed by cells migration from the inner to the outer layers^32,33^). Results showed that the laminar structure of the retina and its general architecture are morphologically indistinguishable between control and *Bmal1*cKO (Fig. 2a,b). INL and ONL thicknesses were measured on DAPI-stained retinal slices, and no statistically significant differences between genotypes were found (Fig. 2c; n = 4/4 Ctrl and *Bmal1*cKO; Total retinal thickness:167.20 ± 7.00 μm vs. 175.34 ± 2.85 μm, INL 28.69 ± 0.99 μm vs. 32.12 ± 1.26 μm and ONL 67.58 ± 2.97 μm vs. 69.25 ± 1.70 μm, respectively in Ctrl vs. *Bmal1*cKO retinae; P > 0.05, Multiple *t*-tests). Moreover, it is also known that, in the retina, the stress of any origin induces an immediate reactive response of Müller cells that orchestrate downstream events such as activation of self-protective mechanism (fibroblast growth factor 2, FGF-2) and activation of microglial cells. Hence, we analyzed Ctrl and *Bmal1*cKO retinal sections immuno-labeled for glial fibrillary acidic protein (GFAP)—whose expression in Müller cells is known as a hallmark of early gliosis^34^—and Ionized calcium-binding adaptor molecule 1 (IBA1), a marker of microglial cells. Results revealed no significant differences in GFAP expression level (Fig. 2e) and its localization in the retinal nerve fiber layer (Fig. 2b), as well as no differences in the number of microglia and their localization within retinal layers (Fig. 2b,d; n = 8/8 Ctrl and *Bmal1*cKO; GCL + IPL: 6.76 ± 0.29 vs. 7.29 ± 0.26, INL + OPL:6.03 ± 0.41 vs. 6.66 ± 0.35 and ONL:0.52 ± 0.15 vs. 0.35 ± 0.15, respectively in Ctrl vs. *Bmal1*cKO per 1000 μm segment of the retina; P > 0.05, Student’s *t*-test).

Our data indicate that conditional deletion of *Bmal1* in glial cells through the Cre-loxP system and TM treatment did not cause any detectable structural change or glial reactivity. Therefore, we decided to address whether clock gene expression-related changes could drive the observed functional effects of *Bmal1* deletion on the ERG photopic response. Moreover, since photopic light-adapted ERG reflects cone-mediated light responses, our data suggest an altered transmission of information from cones to the bipolar cells in the *Bmal1*cKO mice retina.

### Loss of glial *Bmal1* globally alters clock gene expression rhythms in the retina in the LD cycle

**Figure 3:**
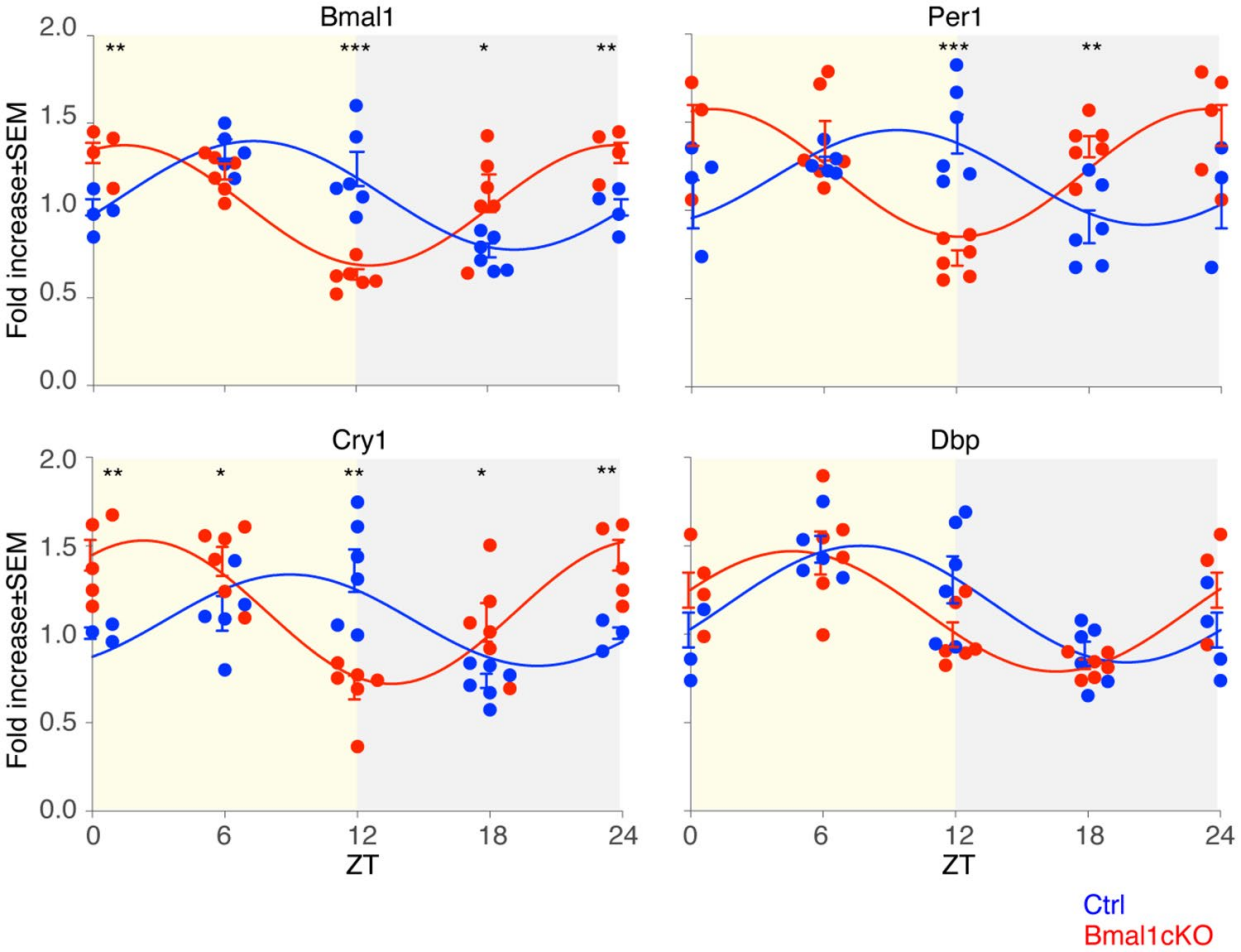
Altered rhythmic clock-genes expression in the retina upon GLAST^+^ cells-specific *Bmal1* deletion. Altered daily rhythms expression of some clock genes, i.e., *Bmal1*, *Cry1*, *Per1,* and *Dbp,* in the retina of *Bmal1*cKO (red) compared to Ctrl (blue) in adult mice under 12:12 h LD cycles. The yellow background depicts the light phase (ZT 0–12), and the gray represents the dark phase (ZT 12–24). Samples were harvested at four equally distributed time points during the day, and ZT24 time points are the ZT0 ones shown again. Each curve depicts the best fitting (P < 0.05) between a cosine fitting with a period of 24 h or linear regression. Mean ± SEM of 4–6 animals per group at each time point, performed in triplicates (n = 4–6 per strain, Multiple t-tests, *P < 0.05, **P < 0.01, and ***P < 0.001 vs. Ctrl) are shown.

We determined the impact of the adult glia-selective deletion of *Bmal1* on the core clock gene oscillations in the global retina, consistent with a role for glial clock gene networks in setting the intrinsic circadian cycle of the retina. Transcript levels of clock genes were measured over a daily cycle of 12 h of light and 12 h of dark (LD, light–dark) using quantitative real-time PCR (qPCR) in retinae from adult Ctrl and *Bmal1*cKO mice two months after TM treatment. Samples were harvested at four equally distributed time points during the day (ZT, Zeitgeber Time). Consistent with previous reports^11,35^, *Bmal1*, *Cry1*, *Per1,* and the clock-regulated gene *Dbp*, which expression is under the direct control of CLOCK-BMAL1 activity^36^, were rhythmically expressed in control mice retinae in the 12:12 h LD condition (Fig. 3; n = 4–6/4–6 Ctrl and *Bmal1*cKO). Oscillation amplitudes in retinae from control animals were compatible with those previously reported in this tissue^37,38^. In particular, as formerly described^35–37^, *Per1* and *Cry1* mRNA reached their maximum expression levels at ZT12, while *Bmal1* and *Dbp* peaked at around ZT6 in controls (Fig. 3, blue).

In contrast, the deletion of *Bmal1* in GLAST^+^ cells advanced the acrophase of all analyzed core clock gene transcripts in the whole retinal tissue, except for the BMAL1 target *Dbp*, however it did not affect their amplitudes (Fig. 3, red). The similar oscillation of *Dbp* between controls and *Bmal1*cKO mice retinas (Fig. 3, bottom right) indicates that compensatory mechanisms might account for its rhythm or that some retinal cells might have retained their rhythmic expression. Altogether, our results show that the loss of the *Bmal1* gene in retinal astrocytes and Müller cells alters the overall retinal circadian clock gene expression by changing the timing of the expression peaks. This observation expands the evidence of the critical interplay of neuronal and glial cells in regulating global molecular clock expression within the retina, in agreement with results observed in other tissues such as the cortex, hippocampus, and SCN^15,17,39^.

### Functional heterogeneity within the RGCs population is not altered in the *Bmal1cKO* mice retina

Retinal ganglion cells (RGCs) represent the output neurons of the retina that provide spike-train encoded visual information to downstream brain circuits. Given the dysfunctional retinal response to light assessed with the in vivo ERGs and the altered phasing of circadian clock genes in the retina of *Bmal1*cKO mice, we wondered whether these changes might impact the retinal output at the level of light-evoked RGCs responses^40^.

To evaluate the effects of glial *Bmal1* deletion on the retinal functional output, we first investigated whether RGCs retain their light response and could be classified according to their polarity preference as ON, ON–OFF, and OFF RGCs. We used a 4096 electrodes CMOS high-density multielectrode array (CMOS HD-MEA, 3Brain AG, Wädenswil, Switzerland) to record light-evoked activity in response to full-field flash stimuli presented to Ctrl and *Bmal1*cKO mice retinae under different Michelson contrast conditions (CT25, 50, 75 and 100, see Material and Methods) and in both mesopic and photopic light regimes.

**Figure 4:**
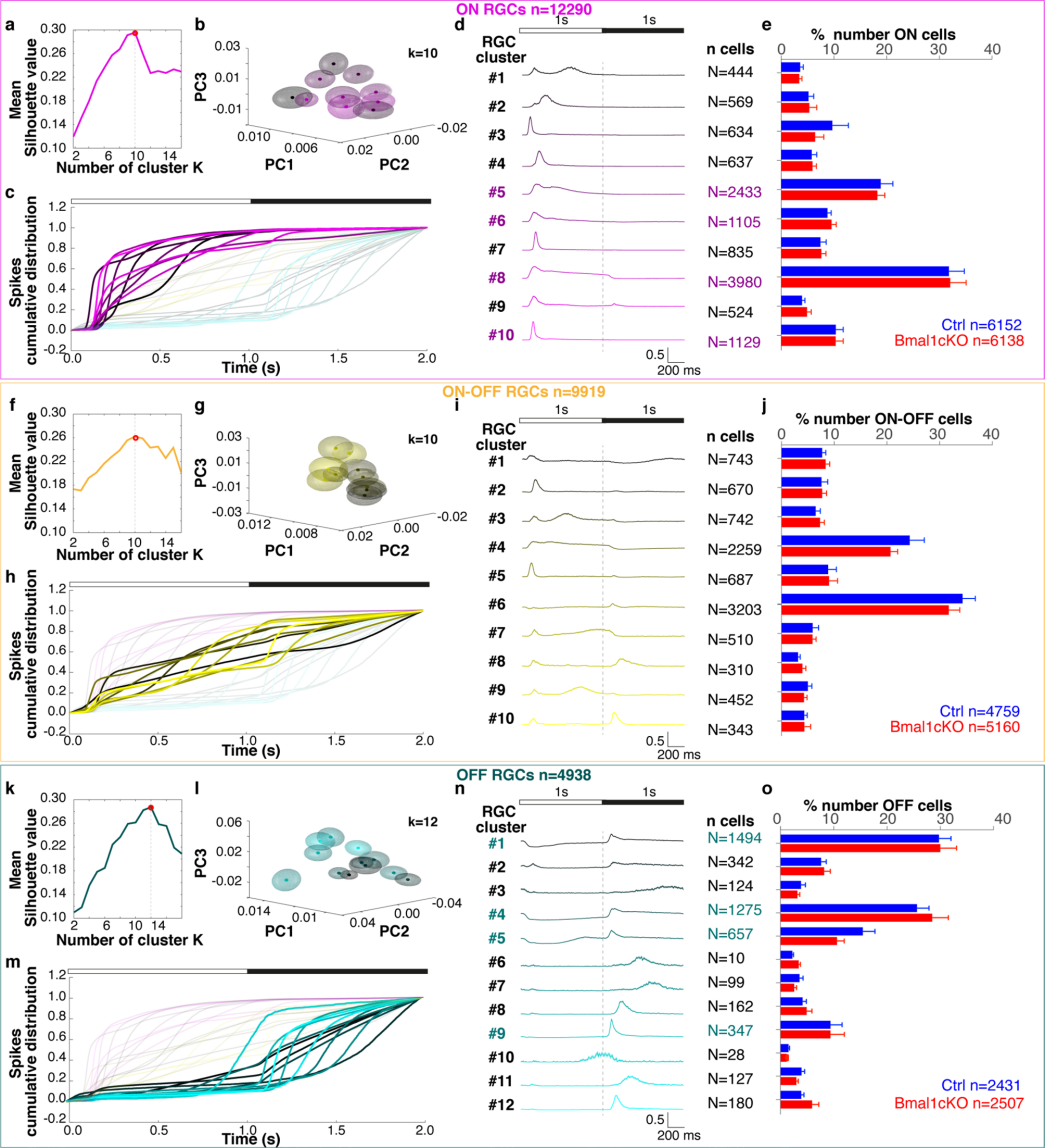
Functional characterization of ON, ON–OFF and OFF type RGCs detected from responses to full-field flashes in Ctrl and *Bmal1*cKO retinae under mesopic conditions. **(a–f–k)** The mean Silhouette analysis (average silhouette values 0.30, 0.26, and 0.28 for ON, ON–OFF, and OFF RGCs, respectively) suggests an optimal number of 10 clusters for ON and ON–OFF RGCs, and 12 for OFF RGCs. “k” represents the number of clusters that maximize the silhouette width. **(b–g–l)** Principal component analysis (PCA) of ON, ON–OFF, and OFF RGCs. **(c-h-m)** Cumulative spike trains distribution to alternating 1 s white and 1 s black flashes (20 repetitions, CT100) for ON, ON–OFF, and OFF RGCs. Each line represents the averaged response of the cells belonging to the color-coded cluster. **(d–i–n)** Population response for ON, ON–OFF, and OFF cells (10 ON, 10 ON–OFF, and 12 OFF clusters) per cluster. Each row shows the normalized PSTH of a single cluster. The numbers on the right of the PSTHs represent the numerosity of each cluster. **(e–j–o)** The histograms denote the relative proportion of recorded RGCs from each retina (Ctrl in blue and *Bmal1*cKO in red) assigned to the cluster. Cluster numerosity does not exhibit bias toward one of the two strains (Multiple *t*-tests correct). Data are shown as mean ± SEM. On the bottom right of each panel, the total amount of ON, ON–OFF, and OFF RGCs recorded in Ctrl and *Bmal1*cKO. Clusters ON 5, 8, 6, 10 and clusters OFF 1, 4, 5, and 9 represent the most abundant and further analyzed clusters.

In mesopic conditions, the response of around 27000 single units acquired at 6-h intervals over the day/night cycles (i.e., ZTs 0–6–12–18) from 26 Ctrl and 25 *Bmal1*cKO mice retinae were considered for further analyses. Every unit was classified as ON, ON–OFF or OFF based on the shape of the cumulative distribution spike trains to alternating flashes (1 s white, 1 s black) at maximum contrast using a greedy template matching approach (see Material and Methods)^41^. Accordingly, ON cells preferentially respond to light increments, OFF cells to light decrements, and ON–OFF to both. An average of 241 ± 14 ON, 97 ± 6 OFF, and 194 ± 14 ON–OFF RGCs (mean ± SEM) per experiment were included in the analysis. Since we used a unique set of templates to classify cell polarity in both Ctrl and *Bmal1*cKO retinae, we could pool data across all recordings and cluster RGCs responses to 2 s of light stimulation. In the first step, a k-means unsupervised clustering algorithm based on the cumulative response to the full-field flashing stimuli (Fig. 4c,h,m) was used to assess the degree of light response diversity in overall recorded cells (TOT = 27147 from Ctrl and *Bmal1*cKO mice retinae; ON RGCs n = 12290, ON–OFF RGCs n = 9919, OFF RGCs n = 4938 in mesopic condition). We then used a Silhouette analysis to determine the optimal number of clusters of RGCs functional subtypes in an unsupervised way. Under mesopic conditions at 100% Michelson contrast (CT100), this yielded a total of 32 different RGCs' qualitative responses to full-field stimuli (Fig. 4c,h,m) and the identification (Fig. 4a,b,f,g,k,l) of 10 clusters (k) for ON (pink), 10 clusters for ON–OFF (yellow) and 12 clusters for OFF RGCs (green). The cluster-mean normalized peristimulus time histogram (PSTH) obtained as an average response of all recorded units per cluster to 20 flashes repetitions at CT100 (Fig. 4d,i,n) revealed the presence of known RGCs in the mouse retina^42^.

Similarly, in the photopic condition, we recorded RGCs light-evoked response in a subset of our initial cohort counting 18 Ctrl and 19 *Bmal1*cKO mice retinae (6 Ctrl/5 *Bmal1*cKO ZT0, 4 Ctrl/5 *Bmal1*cKO ZT6, 3 Ctrl/4 *Bmal1*cKO ZT12, 5 Ctrl/5 *Bmal1*cKO ZT18). The contrast preference (ON, ON–OFF, and OFF RGCs) has been measured separately in the photopic condition. We obtained 9 clusters for the ON (TOT = 7755 from Ctrl and *Bmal1*cKO mice retinae; Ctrl n = 4027 and *Bmal1*cKO n = 3748), 8 clusters for the OFF (TOT = 7513 from Ctrl and *Bmal1*cKO mice retinae; Ctrl n = 4010 and *Bmal1*cKO n = 3503), and 10 clusters for the ON–OFF (TOT = 18213 from Ctrl and *Bmal1*cKO mice retinae; Ctrl n = 8855 and *Bmal1*cKO n = 9358) RGC subtypes (see Supplementary Fig. S3 for the functional characterization of ON, ON–OFF and OFF RGCs types in the photopic condition).

Next, to evaluate differences between Ctrl and *Bmal1*cKO retinae, we restricted our analysis to ON and OFF cell types because most OFF RGCs and a large fraction of ON RGCs switch their response preference to ON–OFF type RGCs depending on the stimulus size and ambient luminance^43,44^. To assess the uniformity of RGCs classification across different experiments, we calculated the relative proportions of cells from Ctrl or *Bmal1*cKO mice retinae assigned to each cluster (across different times of the day). Bar plots show that clusters did not exhibit a bias toward one of the genotypes (Fig. 4e,j,o; P > 0.05, Multiple *t*-tests corrected for multiple comparisons using the Holm-Sidak method). This suggests that RGCs retain their characteristic light‐evoked preferential ON and/or OFF responses to full-field flashes and their ability to transmit visual information to central visual areas upon deletion of the glial *Bmal1* clock gene.

### Glial *Bmal1* influences the light response amplitude of RGCs across different times of the day

**Figure 5:**
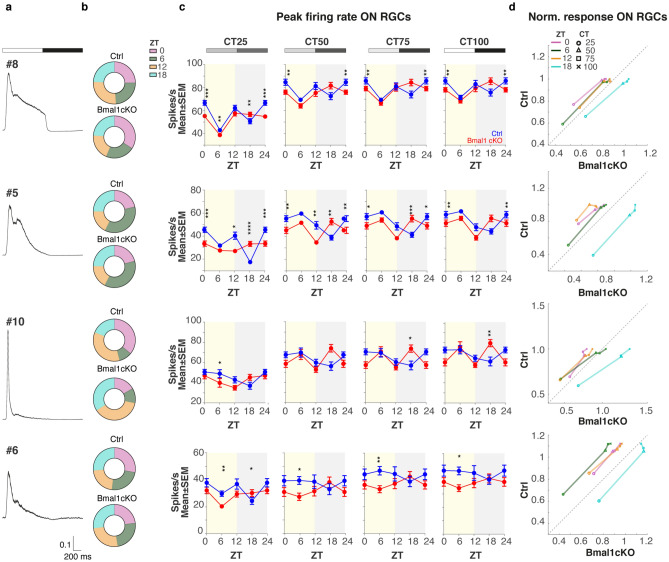
*Bmal1* deletion affects ON RGCs maximum firing rate in response to light in mesopic conditions. **(a)** Averaged normalized PSTH (mean ± SEM) of all RGCs belonging to the most abundant ON clusters (both genotypes, all ZTs at CT100) #8, #5, #10, and #6 in response to full-field white-black alternating flashes (stimulus indicated above). **(b)** The proportion of recorded RGCs per ZTs (Top, Ctrl and bottom, *Bmal1*cKO). All cells from different experiments pulled together. **(c)** Analysis of the maximum firing rate in response to alternating bright and dark flashes performed on selected ON RGCs clusters (different rows) at four different ZTs (0, 6, 12, 18) and different contrasts (CTs, different columns). Data are shown as mean ± SEM between cells belonging to a specific cluster, separately for Ctrl (blue) and *Bmal1*cKO (red). Asterisks mark a significant difference between groups. (Kolmogorov–Smirnov test; *P < 0.05, **P < 0.01, ***P < 0.001, ****P < 0.0001). **(d)** Scatter plots of RGCs peak amplitudes normalized to the maximal response of the control throughout the day in Ctrl (y-axes) vs. *Bmal1*cKO (x-axes) conditions. Different colors indicate results obtained at diverse ZTs, and the symbols denote contrast conditions. Dashed line: unity line.

**Figure 6:**
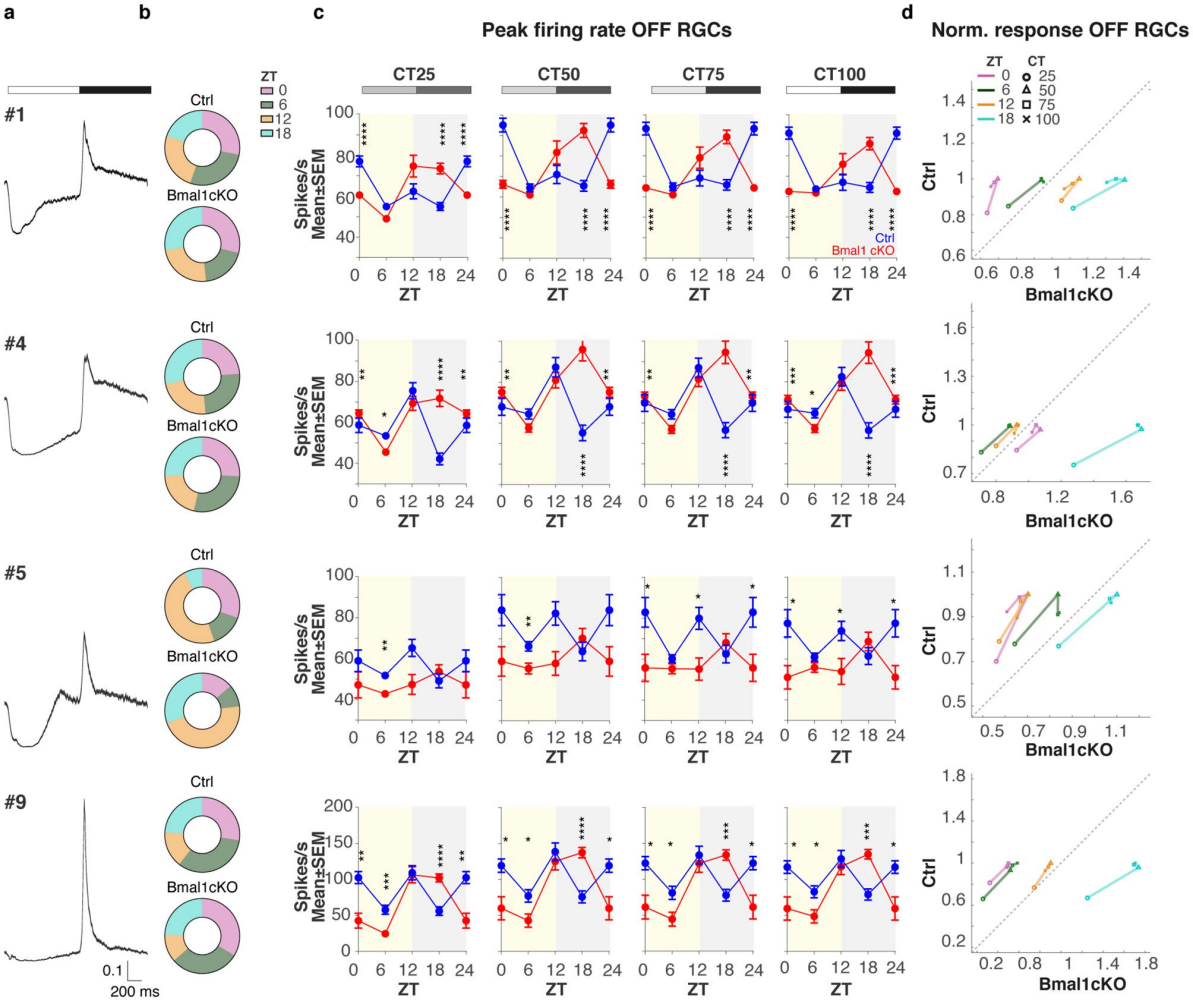
*Bmal1* deletion affects OFF RGCs maximum firing rate in response to light in mesopic conditions. **(a)** Averaged normalized PSTH (mean ± SEM) of all RGCs belonging to the most abundant OFF clusters (both genotypes, all ZTs at CT100) #1, #4, #5, and #9 in response to full-field white-black alternating flashes (stimulus indicated above). **(b)** The proportion of all recorded RGCs per ZTs (Top, Ctrl, and bottom, *Bmal1*cKO). **(c)** Analysis of the maximum firing rate in response to alternating bright and dark flashes performed on selected OFF RGCs clusters (different rows) at four different ZTs (0, 6, 12, 18) and different contrasts (CTs, different columns). Data are shown as mean ± SEM between cells belonging to a specific cluster, separately for Ctrl (blue) and *Bmal1*cKO (red). Asterisks mark a significant difference between groups. (Kolmogorov–Smirnov test; *P < 0.05, **P < 0.01, ***P < 0.001, ****P < 0.0001). **(d)** Scatter plots of RGCs peak amplitudes normalized to the maximal response of the control throughout the day in Ctrl (y-axes) vs. *Bmal1*cKO (x-axes) conditions. Different colors indicate results obtained at diverse ZTs, and the symbols denote contrast conditions. Dashed line: unity line.

We then investigated whether stimulus-driven responses of RGC subtypes, which encode distinct aspects of the visual scene, are affected by day-night cycles and by glial knockout of *Bmal1*. For the four assessed different Zeitgeber times (ZT, i.e., 0, 6, 12, 18; the relative proportions of ON and OFF RGCs sampled at different ZTs in Ctrl and *Bmal1*cKO retinae are reported in Figs. 5b, 6b) and Michelson contrast levels (CT, i.e., 25, 50, 75, 100), we extracted PSTHs of single cells from the four most abundant clusters for both ON and OFF RGCs and we calculated the light-evoked maximum firing rate value (peak of responses). Specifically, we further studied the activity throughout the day of the most basic functional groups, sustained ON RGCs from clusters #8 and #5, and transient ON RGCs from clusters #10 and #6 classified based on the duration of their response (Fig. 5a, averaged PSTHs of all cells for each of the indicated clusters at CT100. See Supplementary Fig. S4a,b for functional ‘fingerprint’ of ON selected cells). Similarly, we chose clusters #1, #4 and #5, #9 for OFF RGCs that show a sustained and transient response to a flashing spot, respectively (Fig. 6a, averaged PSTHs of all cells for each of the indicated clusters at CT100. See Supplementary Fig. S5a,b for functional ‘fingerprint’ of OFF selected cells). Results for ON RGCs responses (Fig. 5c) revealed that the responses to the alternating full-field stimulus of ON sustained RGCs (clusters #8 and #5) in Ctrl conditions were stronger at ZT 0 and ZT 12. In contrast, peak amplitudes for ON transient RGCs (clusters #10 and #6) were more stable over the day (One-way ANOVA with Tukey–Kramer post-hoc correction. Asterisks on the plots refer only to differences between groups). Instead, both sustained and transient analyzed OFF RGCs (Fig. 6c) in Ctrl exhibited a daily variation in light sensitivity. Those features did not vary with the stimulus contrast (CTs) and were altered only in the *Bmal1*cKO group. Overall, the comparison of the peak amplitude averaged across cells and normalized to the maximal response over the day in the control condition for both ON and OFF RGCs, clearly revealed a more prominent light response in *Bmal1*cKO RGCs at ZT18 (Figs. 5c and 6c; Figs. 5d and 6d, cyan curve (ZT18, *Bmal1*cKO vs. Ctrl) below the unity line), indicating a higher sensitivity during nighttime in *Bmal1*cKO retinae. Presenting the stimuli at different contrasts did not affect this outcome. These results indicate that the response amplitude of RGCs sub-types (i.e., cluster of cells with similar spike trains cumulative distribution) is influenced by glial *Bmal1* across different times of the day, suggesting there may be circuit-specific contributions of retinal glia to the circadian regulation of visual processing.

### Glial *Bmal1* effects on luminance dependent RGCs polarity preference

Our ERG data showed that deletion of the core clock gene *Bmal1* in astrocytes and Müller cells alters cone ERG in the light-adapted condition but not in the dark-adapted one. This result aligns with previous reports that showed a tendency for cone-isolating conditions to intensify the effects of the intrinsic retinal circadian clocks on retinal function^11,14^. However, single-unit recordings from a large population of retinal ganglion cells revealed that in mesopic conditions, the glial *Bmal1* deficiency is sufficient to alter the daily modulation of the maximal response to a flash of light in some RGCs clusters, but not the heterogeneity of RGCs physiological subtypes. Given that most OFF-type RGCs and a large fraction of ON-type RGCs switch their response preference depending on stimulus size and ambient luminance^43,44^, we investigated whether this shift was altered as a function of ambient light levels in *Bmal1*cKO compared to Ctrl retinae.

**Figure 7:**
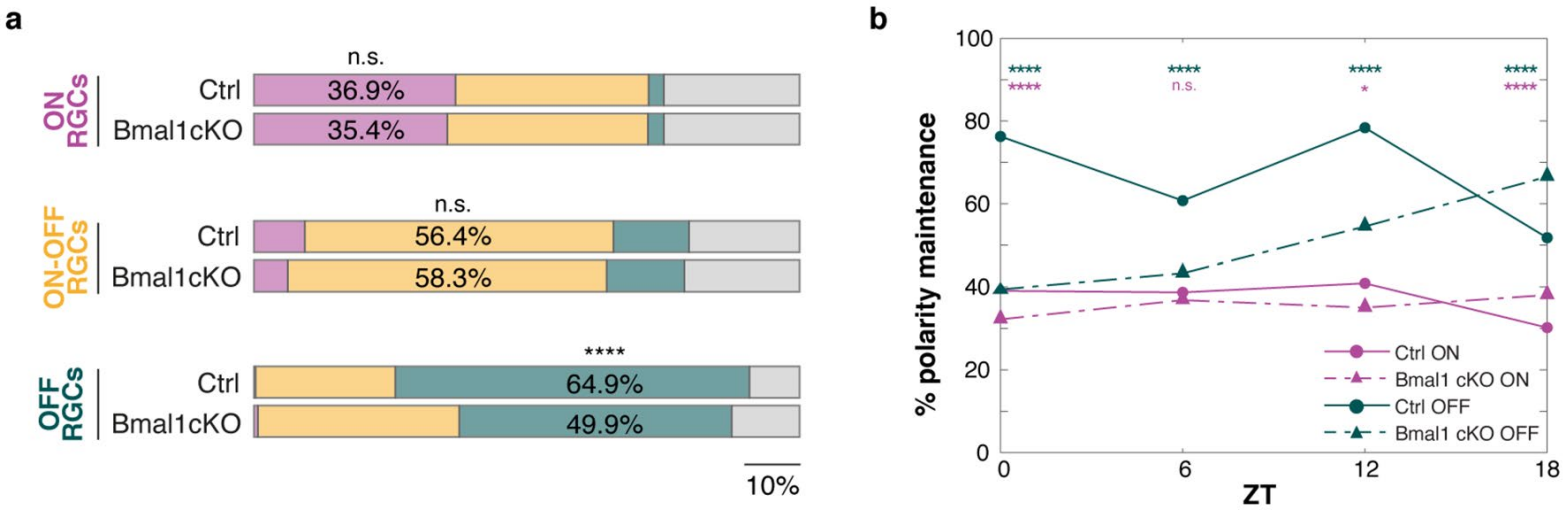
Mean light luminance polarity shift for OFF preferential RGCs is altered upon glial BMAL1 deletion. **(a)** Polarity maintenance was calculated as the percentage of cells within the ON, ON–OFF, and OFF subtypes that did not change their polarity preference at different ambient illumination in Ctrl and *Bmal1*cKO (Fisher’s exact test to assess the difference between groups). **(b)** The analysis in (**a**) has been performed only for ON and OFF RGCs at different ZTs collected from 37 datasets. Significance asterisks are displayed for Ctrl and *Bmal1*cKO comparison. *P < 0.05; ****P < 0.0001; ns = not significant.

Hence, we analyzed the polarity preference of 16515 single-unit RGCs identified from 37 datasets collected from CMOS HD-MEA recordings under both mesopic and photopic regimes (Fig. 7). A similar amount of RGCs in Ctrl and *Bmal1*cKO retinae kept their polarity preference as ON RGCs with increasing luminance (Fig. 7a, 36.9%, and 35.4%, respectively; Fisher’s exact test n.s.). Interestingly, the fraction of OFF RGCs identified in the mesopic condition that switched their polarity preference in the photopic regime was higher in *Bmal1*cKO than Ctrl retinae (Fig. 7a, 49.9% *Bmal1*cKO vs. 64.9% Ctrl OFF RGCs kept their OFF polarity preference; Fisher’s exact test, P < 0.05).

We then investigated whether these luminance-depended shifts in contrast preference are under day-night cycle regulation and to which extent they might be affected by the glial *Bmal1* conditional deletion. Since more than 50% of ON–OFF RGCs showed no transitions in polarity with increasing luminance in both Ctrl and *Bmal1*cKO retinae, we restricted our analysis to ON and OFF RGCs. This analysis revealed that OFF RGCs not only varied their contrast switching behavior at different times, but the “polarity switching profile” is different between Ctrl and *Bmal1*cKO groups (Fig. 7b). Indeed, while the polarity switching profile of OFF RGCs in controls oscillated over the 24-h day (overall 64.9% kept their preferential response), the *Bmal1*cKO OFF RGCs were shifting more their contrast preference at ZT0 being more stable at ZT18 (Fig. 7b). These findings suggested the involvement of glial *Bmal1* in tuning day/night RGCs contrast preference when responding to full-field flash stimuli in different light conditions.

## Discussion

This study shows that the glial clock controls the intrinsic retinal circadian machinery and contributes to the daily variation of retinal physiology. In agreement with previous studies that show the presence of a circadian tuning of photopic and mesopic responses^45^, our results indicate, for the first time, the involvement of glial cells (astrocytes and Müller cells) in the regulation of retinal function throughout the day.

The entire cellular population of neurons and glia in the retina express all canonical clock genes except rod photoreceptors^46^. Accordingly, the original paper from Storch and colleagues^11^ showed the critical role of the core clock gene *Bmal1* in the retinal global circadian clock and function by deleting this gene from all retinal cells since development. Surprisingly, our results revealed that selective adult *Bmal1* deletion in glial cells is sufficient to modify the clock genes expression temporal profile (not their expression level) in the whole retina and to reduce the b-wave amplitude of photopic ERGs, as we observed through light-adapted fERG^45^. Notably, the deletion of *Bmal1* in all retinal cells^11^ and the selective deletion of other clock genes such as *Cry1/Cry2* do not lead to detectable retinal morphological alterations^14,47,48^. Consistently, in our work, the selective deletion of *Bmal1* in GLAST^+^ cells of adult mice retinae did not induce either any noticeable morphological alterations or inflammatory processes that could explain an altered retinal function. Conversely, photoreceptor viability during aging is reduced in *Bmal1*^-/-^ and *Npas2/Clock* double KO mice^49,50^. In line with this finding, subtle morphological alterations have been described in *Per1/Per2* mutants up to 1 year of age^51^.

The reduced fERG response that we observed in the light-adapted cone-isolating condition is in accordance with many studies that have demonstrated the presence of a unique circadian clockwork expressed in cones but not in rods^46,50^. Additionally, it is known that retinal circadian clocks control the strength and the extent of rod-cone electrical coupling (gap junctions) by activating dopamine D2-like receptors^52^. Our fERG results suggest that rod-cone electrical coupling might be dysregulated due to altered clock gene oscillations in *Bmal1*cKO retinae, thus implying that glial *Bmal1* function is required to preserve proper cone light responses.

Although fERG has been primarily used for the electrophysiological assessment of retinal circadian function, it mainly reflects photoreceptors and bipolar cell activity^45^. It does not evaluate implications on the retinal functional output. Thus, to study the possible involvement of glial *Bmal1* in the daily tuning of the ultimate output signals of the retina, we focused our attention on the population of retinal ganglion cells that integrate presynaptic information and carry them out to down-streamed brain areas. Analyzing the response to light stimuli of hundreds of RGCs enabled us to study the functional behavior of major RGCs subtypes at different times-of-the-day and under mesopic and photopic conditions, in both *Bmal1*cKO and Ctrl retinae. Our data revealed that glial *Bmal1* directly or indirectly regulates the daily variation of the peak response amplitude of RGCs subtypes.

In contrast, functional characterization of ON, ON–OFF, and OFF subtypes is preserved under glial *Bmal1* deletion. Indeed, we observed a more robust response to the preferred stimulus in *Bmal1*cKO, both ON and OFF RGCs during the nighttime (ZT18), which indicates a disruption in controlling the sensitivity of the RGCs in *Bmal1*cKO. This observation unveils the role of glial *Bmal1* in regulating visual information processing in the absence of environmental light. A possible explanation for this effect could be related to the relevant role of glia (mainly by Müller cells) on the fine control of the extracellular concentration of ions and neurotransmitters that might be dysregulated in GLAST^+^ *Bmal1*cKO retinae^19,20,26^. It is well known that the retina relies on a complex day-night rhythm to regulate various functions that range from gene expression to fundamental metabolic activities and optimize visual processing in the retina^47,48^. The involvement of glial *Bmal1* in the optimization of visual processing is also supported by our results on the “switching of RGCs polarity preference” with ambient luminance conditions. Our results clued that while the percentage of ON cells that keep their polarity preference in mesopic and photopic conditions is comparable across different times of the day in Ctrl and *Bmal1*cKO retinae, the deletion of glial *Bmal1* significantly influences the contrast preference of OFF RGCs when increasing light luminance.

Overall, this work argues for fine daily control of RGCs light responses, modulated by glial cells, most likely involving Müller cells that act at different levels in the retina, contributing to a specific outcome at different times-of-the-day. Circadian control of RGCs firing rate has been previously suggested only for ipRGCs^53^ and in chronic recordings from the retina in awake mice^54^. Recent data have shown how the rod bipolar-AII amacrine cell circuitry includes a newly discovered GABAergic interneuron which provides a robust inhibitory input to the AII, probably to optimize its role in transmitting weak sensory inputs^55^. Conversely, a glycinergic amacrine cell was strongly linked to Müller cells^56^, amacrine, bipolar, and ganglion cells. Together, these data provide evidence of a robust inhibitory control that links Müller cells with the retinal circuitry, probably keeping AII amacrine cells in a pivotal position. In this respect, looking at the fundamental role played by Müller cells in the control of retinal function and homeostasis^25^ and their peculiar morphological organization^57^, it seems reasonable to hypothesize that most of the effects revealed by the present study might be mediated by Müller cells. This hypothesis opens fascinating perspectives for future studies aimed at clarifying the functional adjustments of retinal processing during the day-night cycle, a topic in which the present study reveals a previously unknown role of glial cells.

## Materials and methods

### Ethical statement

Experiments presented herein were performed at the Italian Institute of Technology (IIT). All the experiments were performed according to the guidelines approved by the European Community Council (Directive 2010/63/EU of September 22, 2010). All experimental animal procedures were approved by the institutional IIT Ethics Committee and the Italian Ministry of Health and Animal Care (Authorization number 110/2014-PR, December 19, 2014). Moreover, all methods were performed by the ARRIVE guidelines.

### Animal model

Conditional deletion of *Bmal1* in astrocytes was achieved as previously described by a Tamoxifen (TM)-inducible knockout mouse model (*Glast*-CreER^T2^; *Bmal1*^*f*lx/flx^ here referred to as *Bmal1*cKO)^17,28^. Briefly, CreER^T2^-recombinase is expressed under the control of the astrocyte-specific glutamate transporter *Glast* promoter^27^. Mice were housed with ad libitum access to food and water and kept on a standard 12 h:12 h light–dark (LD) cycle. White light at 300 lx was presented from zeitgeber time 0 (ZT0, 7 AM) to 12 (ZT12, 7 PM) in a room maintained at 21 °C. *Glast*-CreER^T2^; *Bmal1*^*f*lx/flx^, and controls *Bmal1*^*f*lx/flx^ were both treated with TM dissolved in corn oil at weeks 6–8. Animals received 5 mg per day for two consecutive days by oral gavage. A third treatment was repeated one week before the experiments (Fig. 1a). All studies were performed between 18 and 22 wks. This study includes both male and female mice. *Glast*-CreER^T2^; *Bmal1* WT td-Tomato mice were used to confirm and visualize Cre recombinase activity.

### Tissue processing, morphological analyses, and immunohistochemistry

Mice were euthanized by cervical dislocation after inhaling carbon dioxide (CO_2_), and collected eyes were fixed in 4% paraformaldehyde for 1 h at 4 °C for tissue staining. They were cryoprotected by immersion in 15% and 30% sucrose solution overnight, embedded in the OCT compound (Tissue Tek; Qiagen, Valencia, CA), and frozen. Cross-sections of 18 μm thickness were made for each retina, collected on gelatin poly-l-lysine coated slides, and stored at −20 °C until processed. The analyses were performed on the sections crossing the optic nerve in the dorsal–ventral direction (~ 8 to 10 central slices per retina were collected on different slides) to minimize the retinal length and position variations. Instead, whole-mount isolated retinae were fixed for a further 15 min and washed in PBS before being processed for immunostaining. Retinal slices and whole mounts were permeabilized with Triton X-100 in PBS, blocked with goat serum in PBS, and incubated at 4 °C overnight with the primary antibody [mouse anti-GFAP (SYSY, 173011); rabbit anti-Iba1 (SYSY, 234003); mouse anti-GS (Millipore, MAB302)]. The following day, sections were extensively washed and incubated for 45 min with Alexa-488, -546 secondary antibodies used at 1:1000 dilution (Invitrogen). Nuclei were counterstained with DAPI. Slices were washed, mounted, and imaged in an inverted laser scanning confocal microscope [TCS SP5 microscope using a 20× or 40× objective (Leica Microsystems, Wetzlar, Germany)], and acquisition parameters were kept constant throughout each imaging session. Only one of the two eyes was used for a single marker or histological analysis. Quantification and analysis were performed in ImageJ Fiji software (Rasband, W.S., ImageJ, U. S. National Institutes of Health, Bethesda, MD, USA)^58^.

The morphological analysis was carried out by measuring the absolute thickness (in μm) of the outer nuclear layer (ONL), the inner nuclear layer (INL), and the total retinal thickness across the full extension from dorsal to ventral crossing the papilla. Shortly, histological reconstructions were obtained by joining consecutive acquired micrographs using a 20× objective. To facilitate comparison between retinas, each retina was divided into 10 dorsal and 10 ventral fields while taking the optic nerve as a reference. The layer thickness was calculated as the averages of four equispaced measurements from each of the 20 fields (to account for slight differences within the same area). The averages of the thickness measured in the 10 dorsal or ventral fields were calculated for each section. Moreover, retinal sections were stained with Hematoxylin&Eosin and examined on a Neurolucida microscope equipped with the software Neurolucida (MicroBrightField) and 10× and 20× objectives.

Spatial distribution and the total number of Iba1^+^ microglial cells were counted in the ganglion cell layer and inner plexiform layer (GCL + IPL), in the inner nuclear layer and outer plexiform layer (INL + OPL), and the outer nuclear layer (ONL), respectively. The number of Iba1^+^ cells was counted throughout the entire section from dorsal to ventral (10 images per retinal section acquired at 40× magnification, each long ~ 380 μm). Results are given as the total number of microglial cells (Iba1^+^)/1000 μm retinal segment. Each data point represents the average obtained from two sections per retina. Densitometric analysis of GFAP fluorescent signal over all retinal layers is represented as the average obtained from two sections per retina.

### PCR for genotyping and animal model validation

Mouse tail DNA was extracted and amplified through Kit Thermo Scientific Phire Tissue Direct PCR Master Mix for PCR genotyping. The following primers were used for the Cre transgene as described in^27^:

*GLAST-CRE ERT2-F8* 5′-GAGGCACTTGGCTAGGCTCTGAGGA-3′

*GLAST-CRE ERT2-R3 * 5′-GAGGAGA TCCTGACCGA TCAGTTGG-3′

*GLAST-CRE ERT2-CER* 5′-GGTGTACGGTCAGTAAATTGGACAT-3′

Whereas genotyping for the *Bmal1*^flx/flx^ locus was performed by using the method described in^11^ :

*Forward (oIMR7525) Int8/9* 5′-ACTGGA AGTAACTTTATCAAACTG-3′.

*Reverse (oIMR7526) Int8/9* 5′-CTGACCAACTTGCTAACAATTA-3′.

6–8 weeks after TM treatment, gDNA extracted from different tissues (retina, ON, and cerebellum) was amplified to verify that the deletion occurred. Forward primer for *Bmal1 Int7/8* (5′-GGGTGGAGTATGATATGACC-3′) was designed and used with the reverse primer *Int8/9*; the thermic profile (60 s at 98 °C for DNA polymerase activation, followed by 35 cycles of 5 s at 98 °C for template denaturation, 5 s at 55 °C for primer annealing and 7 s (Supplementary Fig. S2, left panel) or 20 s (Fig. 1b and Supplementary Fig. S2, right panel) at 72 °C for amplification) was adapted to obtain the ~ 2kbp and 0.27kbp bands relative to wild type and disruption of Bmal1 conditional allele. Moreover, primary Müller cells were isolated and cultured from controls and *Bmal1*cKO adult retinas after TM treatment, as previously described by^59^. Briefly, to isolate Müller cells, retinas were removed and incubated in a digestion buffer (with papain and DNase solution) at 37 °C for 5 min. Pelleted cells were resuspended in DMEM with 10% fetal bovine serum (FBS) and 1% Pen/strep antibiotics, cultured in gelatin-coated dishes, and maintained at 37 °C in a 5% CO_2_ incubator. Adherent cells were identified by their morphology and GS immunostaining (Fig. 1c). Total RNA was extracted and retrotranscribed to cDNA (see Material and methods “RNA isolation and real-time qRT-PCR studies”). The following primers *forward Ex6/7* 5′-TGACCCTCATGGAAGGTTAGAA-3′ and *reverse Ex9/10* 5′-GCTGCCCTGAGAAATTAGGTGTT-3′ were designed to get both the 300 bp and 142 bp band relative to wild type and knocked out cells (Fig. 1c). Phire Tissue Direct PCR Master Mix contains a premixed gel loading dye. After PCR, samples were loaded on the electrophoresis gel for analysis, exposed, and photographed in an ImageQuant LAS 4000 mini (GE Healthcare Bio-Sciences AB).

### RNA isolation and real-time qRT-PCR studies

Retinal tissues were collected for RNA isolation at the ZTs 0–6–12–18 from animals in LD cycles (12–12 h). For each time point, at least six animals per group were sacrificed. Total RNA was extracted using TRIzol reagent (Life Technologies) following the manufacturer’s instructions. RNA was further treated to remove gDNA (DNase I AMPD1-1KT, Sigma), and its integrity and quantity were determined by a Nanodrop ND1000 microspectrophotometer (Thermo Fisher Scientific). Complementary DNA was obtained by retro transcription of 500 ng of total mRNA using the M-MulV-RH First-strand cDNA Synthesis Kit (Experteam) following the manufacturer’s instructions. The expression of specific mRNAs was determined by real-time reverse transcriptase–PCR using iTaq Universal SYBR green supermix (BioRad). The gene expression levels were calculated by the ∆∆Ct method based on the control ZT0 gene expression. For a 10 $\upmu$l reaction, 5 ng of cDNA template was mixed with the primers to a final concentration of 400 nM and 5 μl of 2× iTaq Universal SYBR Green Supermix. The reactions were done in triplicates using the following thermic profile: 30 s at 95 °C for DNA polymerase activation, followed by 40 cycles of 15 s at 95 °C, and 60 s at 60 °C for amplification. At the end of the assay, a melting curve was constructed to verify the specificity of the reaction. Glyceraldehyde-3-phosphate dehydrogenase (*Gapdh*) transcripts level was used as reference controls (housekeeping). Sequences of primers are available upon request.

### Electrophysiological recordings

#### In vivo retina recording (fERG)

Flash electroretinograms (fERGs) were recorded during mid-day hours (ZT6) to investigate retinal electrical activity in vivo in an experimental condition described by Barnard et al. as “day-dark” i.e., tested during the day, but light not turned on^13^. Mice were dark-adapted overnight (from 7 PM to avoid circadian rhythm alteration), and electroretinograms were recorded in a completely darkened room. Briefly, animals were anesthetized by an intraperitoneal injection of Ketamine/Xylazine (Ketavet 100—Rompun 20 mg/ml Bayer). Corneas were anesthetized with a novocaine drop, and pupils dilated with 1.0% tropicamide. Animals were mounted on a stereotaxic apparatus and positioned inside the opening of the Ganzfeld dome. Body temperature was maintained at 37.0 °C using a heating pad controlled by a rectal temperature probe. Recordings were carried out for both eyes simultaneously, with a platinum electrode ring electrode placed on the corneas. The reference electrodes were inserted subcutaneously in the proximity of the eyes, while the ground electrode was inserted in the anterior scalp, between the eyes. The standard ERG protocol advocated by the ISCEV (International Society for Clinical Electrophysiology of Vision) was used^60^. Mice were adapted to a background illumination (17 cd s/m^2^, 10 min) before recording responses at progressively brighter short flashes (0.001–30 cd s/m^2^ range) over 450 ms, plus 50 ms pre-trial baseline. Responses were averaged (3 per luminance) to reduce variability and noise, with an interstimulus interval ranging from the 60 s for lower intensities to 5 min (dark-adapted ERG). Light-adapted ERG consisting of 20 replicate responses elicited by 1 Hz flashes of white light (30 cd s/m^2^, photopic condition) was subsequently performed. Responses were amplified differentially, filtered in the 0.2–5000 Hz frequency band, and sampled at 16.3 kHz. Custom-written procedures in IGOR Pro 6.3 (Wavemetrics, Lake Oswego, OR, USA) software was used to analyze the electrophysiological data recorded. We evaluated the a-wave amplitude as the first negative deflection and b-wave amplitude, from the a-wave peak to the positive b-wave peak (in μV). The distributions of ERG responses are described by means and standard errors. Data were assessed for normality using the Shapiro–Wilk test, with the subsequent assessment for statistical significance using the two-tailed Student’s *t*-test (for normally distributed data).

### Ex vivo retina recording (HD-MEAs)

#### Tissue preparation for electrophysiology

Mice (7 Ctrl/5 *Bmal1*cKO ZT0, 8 Ctrl/8 *Bmal1*cKO ZT6, 6 Ctrl/6 *Bmal1*cKO ZT12, 5 Ctrl/6 *Bmal1*cKO ZT18, Tot = 51 mice) were kept in dark-adapted condition for 1 h. Mice were euthanized by cervical dislocation after inhaling carbon dioxide (CO_2_) at different ZTs to examine time-of-day differences in the RGCs activity. As described in^61^ and^62^, eyes were immediately enucleated and dissected in a Petri dish containing AMES medium (Sigma, Merck KGaA, Darmstadt, Germany) supplemented with 1.9 g/L of sodium bicarbonate equilibrated with carboxygen (95% O_2_ and 5% CO_2_). Upon eyeballs enucleation, the cornea, crystalline, sclera, and vitreous were accurately removed to isolate the retinal tissue under a dissecting microscope. Next, the tissue was placed onto a CMOS-MEA, preconditioned with Neurobasal for two hours at 37 °C, with the retinal ganglion cells (RGCs) layer facing the electrodes, leaving the photoreceptor layer exposed to light. A polyester filter (Sterlitech Corp., Kent, WA, USA) and a circular anchor were used to secure the retina. Retinae were continuously perfused using a peristaltic pump at a flow rate of 4 ml/min with an oxygenated bicarbonate-buffered AMES medium during experiments. Data acquisition started about 45 min after the retina was placed in the chamber to let the spikes' amplitude be stable. All procedures were performed in dim red light, and the room was dark throughout the experiment.

### Experimental setup and visual stimulation protocols

Multi-electrode array recordings of RGCs were performed on isolated mice retinas while projecting light stimuli onto the photoreceptors. The setup includes a custom-made photostimulation system based on a digital light projector that generates stimuli at a 60 Hz refresh rate (DLP, LightCrafter Evaluation Module, Texas Instruments), an optical part to project images (664 × 664 pixels, each light pixel covers 4 × 4 μm^2^ of the active chip area) on the tissue lying on the electrode array and the BioCam4096 platform with 4096 Arena chips (3Brain AG, Wädenswil, Switzerland). This platform allows simultaneous recording from 4096 electrodes (21 × 21 μm^2^ size, pitch 42 μm), resulting in a total active area of 2.67 × 2.67 mm^2^. Full-array recordings, sampled at a frequency of 7.022 kHz/electrode, were digitized at a 12-bit resolution per electrode and sent to an offline processing unit upon applying a low-pass filter using Brainwave software.

Stimulus intensities were adjusted using neutral density filters to attenuate light intensity presented to the retina, obtaining a final mesopic (irradiance 0.0134 μW/cm^2^ or 0.092 cd/m^2^) and photopic (irradiance 1.34 μW/cm^2^ or 9.20 cd/m^2^) luminance at maximum brightness^63–65^, with both rods and cones contributing to the response. 26 Ctrl and 25 *Bmal1*cKO retinae (7 Ctrl/5 *Bmal1*cKO ZT0, 8 Ctrl/8 *Bmal1*cKO ZT6, 6 Ctrl/6 *Bmal1*cKO ZT12, 5 Ctrl/6 *Bmal1*cKO ZT18) were recorded in the mesopic condition. Instead, for only 18 Ctrl and 19 *Bmal1*cKO (6Ctrl/5 *Bmal1*cKO ZT0, 4 Ctrl/5 *Bmal1*cKO ZT6, 3 Ctrl/4 *Bmal1*cKO ZT12, 5 Ctrl/5 *Bmal1*cKO ZT18), the same set of stimuli was presented at both luminance levels.

Light stimulation consisted of 5 min of static mean gray background to record spontaneous baseline activity, which we used to determine the reference firing regime of RGCs before presenting any stimulus. A sequence of 60 s mean gray (initial baseline) and 20 trials of full-field light flashes alternating every 1 s from dim to bright at different contrasts symmetrical around the isoluminant gray were recorded. The mean contrast between the darkest (L_min_) and the brightest (L_max_) luminance was quantified through the Michelson contrast convention, defined as 100 × (L_max_—L_min_)/(L_max_ + L_min_), with 100 representing a constant multiplier used to obtain values in the range 0–100. The luminance contrast of the stimuli is referred to as CT, followed by the value obtained in the formula above (e.g., CT25, CT50, CT75, and CT100). The same mean gray background used to assess the basal firing rate was applied to the retina during the time intervals between different stimuli. To synchronize the presentation of the images and light-evoked activity at a sub-millisecond resolution, we used a device that reads the image from the display output and produces a trigger every time the image changes. This information is kept on an electrode channel simultaneously with the extracellular signals recorded on the other electrodes.

### Data processing

#### Spike sorting algorithm

Offline raw data analysis was performed using an automated method to detect and sort the spikes in the extracellular traces to allocate them to single units using the shape and spatial distribution of their waveforms, exploiting the redundant information of neighboring electrodes^66^. Only single units exhibiting at least 0.1 spike/s were considered for subsequent analysis.

#### RGCs polarity preference

The resulting dataset consists of hundreds of single units per retina (532 ± 30, mean ± SEM) characterized as ON, OFF, ON–OFF, and unknown according to their preferential responses elicited by full-field flashes. We restricted our study to cells whose interspike-interval (ISI) distribution for static iso-luminant gray stimuli and white/black flashes at the highest contrast (CT100) differed significantly (Kolmogorov–Smirnov test)^67^. The polarity of the cells was defined by interpreting the spike trains^68^ in response to alternating flashes from white to black as a distribution, intrinsically normalizing all the cells with respect to the number of elicited spikes. Hence, it is straightforward to compare cells firing rates because the method is only sensitive to how spikes are distributed in time rather than their amount. The shapes of the spike train cumulative distributions were matched to a unique set of cell-type-specific templates that defined ON, ON–OFF, or OFF RGCs (template-based matching approach^41^). The contrast preference of RGCs has been separately measured in the mesopic and photopic light levels, considering that RGC’s polarity preference can change across different ambient illumination ranges.

#### Clustering RGCs light-evoked activity

ON, ON–OFF, or OFF RGCs were split into sub-clusters based on their light response properties. An unsupervised parametric free k-means method was applied to cluster RGC light-evoked responses to the highest contrast (CT100) full-field flashing stimulus after pulling them together to be as unbiased, conservative, and transversal as possible across different experimental conditions (ZT, i.e., times of the day) and genotypes (Ctrl vs. *Bmal1*cKO). Specifically, a principal component analysis (PCA) was used to extract features from the normalized spike-trains cumulative distributions of all ON, ON–OFF, or OFF recorded single unit RGCs, separately. Subsequently, the optimal number of clusters was evaluated through a silhouette analysis. Finally, a k-means clustering algorithm subdivides the original dataset into the optimal number of clusters. The clustering was separately performed on the data collected in the two luminance conditions. To validate our results, we calculated the relative abundance of each cluster separately for Ctrl and *Bmal1*cKO averaging results across retinae, such as 100% is given by the total number of ON, ON–OFF or OFF cells per genotype. The same analysis was repeated isolating the experiments according to the time of the day.

### Data analysis

#### Post-stimulus time histogram (PSTH) and peak analysis

After data processing, the instantaneous firing rate per each cell in response to alternating 1 s bright/1 s dark full-field stimuli was quantified by averaging multiple trials. The maximum number of spikes (spikes/sec) elicited after presenting RGCs preferential stimulus (bright stimulus for cells classified as ON and dim for the OFF cells) was calculated using a bin width of 10 ms. Recorded RGCs belonging to different datasets were grouped within each cluster based on their strain (Ctrl and *Bmal1*cKO), ZT (0–6–12–18), and stimulus contrast (CT25–50–75–100): mean and standard error of the mean (SEM) of peak responses was computed. The difference between Ctrl and *Bmal1*cKO cells (within each cluster at a single time point and contrast) was evaluated by computing a Kolmogorov–Smirnov non-parametric test. In comparison, significance over different times of the day within the same group was calculated with a one-way ANOVA followed by a Tukey–Kramer posthoc test.

The same data were represented in scatter plots of normalized responses in control (y-axis) and *Bmal1*cKO (x-axis) conditions. Each data point represents the response to the four contrast steps (different markers) at different ZT (different colors), averaged across cells belonging to a single cluster. The responses are normalized to the maximal response in the control condition (across all the CTs at that ZT).

#### Luminance-dependent polarity maintenance

All RGCs collected from 37 datasets which spike trains cumulative distribution in response to full-field stimulation in both mesopic and photopic regimes matched with one of the templates (see RGCs sub-type classification in ON, ON–OFF, and OFF cell) were collapsed together in a unique dataset composed by 16515 RGCs (446 ± 28, mean ± SEM per dataset), keeping track of the strain and the daytime in which each of them has been collected. If the labeling (i.e., ON, ON–OFF, or OFF preferential) coincided in the two luminance conditions, the cell was considered a cell that maintained its contrast preference; otherwise, it was not. All the RGCs that did not fulfill the initial constraint were discarded into the ‘NA’ (Not Assigned) group. Fisher's exact test compared the percentage of cells that maintained their response polarity in the two conditions.

### Statistical analyses

Statistical parameters, including the exact value of n, mean ± standard error of the mean (SEM), and statistical significance, are reported in the text and figure legends. All statistical tests were two-sided. Statistical comparison of the two groups was made by Student’s *t*-test after assessing for normality using the Shapiro–Wilk test. Otherwise, Kolmogorov–Smirnov non-parametric test was used. A data set fails the normality test when the P-value is ≤ 0.05, meaning it deviates significantly from a normal distribution. The cutoff for significance was P < 0.05, and the significance is marked by *P < 0.05, **P < 0.01, ***P < 0.001, and ****P < 0.0001. As previously reported, the statistical significance of the rhythmic expression was determined by Cosinor analysis^17^. Briefly, for each data set, the fit of a cosine wave (least-squares regression) using the following equation: Y = Baseline + Amplitude × cos (Frequency × X + PhaseShift) was compared with the fit of a horizontal line with the extra sum-of-squares F test. To further assess the goodness of fit of the curves generated from the cosine wave model, the D’Agostino-Pearson omnibus (K2) normality test was applied to the data. Multiple t-tests followed by Holm-Sidak correction were performed to compare the effect of *Bmal1* conditional deletion at each ZT when assessing gene expression. Statistical analyses were performed using GraphPad Prism version 9.0.0 for Windows (GraphPad Software, San Diego, California USA, https://www.graphpad.com) and MATLAB R2018b and Statistics and Machine Learning Toolbox (The MathWorks, Inc., Natick, Massachusetts, United States)^69^.

## Supplementary Information

Supplementary Figures.

## Data Availability

All data and materials used and analyzed during the current study are available from the corresponding author upon reasonable request.

## References

[1] Takahashi JS, Hong H-K, Ko CH, McDearmon EL (2008). The genetics of mammalian circadian order and disorder: Implications for physiology and disease. Nat. Rev. Genet..

[2] Mohawk JA, Green CB, Takahashi JS (2012). Central and peripheral circadian clocks in mammals. Annu. Rev. Neurosci..

[3] Balsalobre A (2002). Clock genes in mammalian peripheral tissues. Cell Tissue Res..

[4] Ramkisoensing A, Meijer JH (2015). Synchronization of biological clock neurons by light and peripheral feedback systems promotes circadian rhythms and health. Front. Neurol..

[5] Sack RL, Lewy AJ, Blood ML, Keith LD, Nakagawa H (1992). Circadian rhythm abnormalities in totally blind people: incidence and clinical significance. J. Clin. Endocrinol. Metab..

[6] Panda S (2002). Melanopsin (Opn4) requirement for normal light-induced circadian phase shifting. Science.

[7] Tosini G, Menaker M (1996). Circadian rhythms in cultured mammalian retina. Science.

[8] Kamphuis W, Cailotto C, Dijk F, Bergen A, Buijs RM (2005). Circadian expression of clock genes and clock-controlled genes in the rat retina. Biochem. Biophys. Res. Commun..

[9] Ruan G-X, Zhang D-Q, Zhou T, Yamazaki S, McMahon DG (2006). Circadian organization of the mammalian retina. Proc. Natl. Acad. Sci. USA.

[10] Sakamoto K (2000). Two circadian oscillatory mechanisms in the mammalian retina. NeuroReport.

[11] Storch K-F (2007). Intrinsic circadian clock of the mammalian retina: importance for retinal processing of visual information. Cell.

[12] McMahon DG, Iuvone PM, Tosini G (2014). Circadian organization of the mammalian retina: From gene regulation to physiology and diseases. Prog. Retin. Eye Res..

[13] Barnard AR, Hattar S, Hankins MW, Lucas RJ (2006). Melanopsin regulates visual processing in the mouse retina. Curr. Biol..

[14] Cameron MA (2008). Electroretinography of wild-type and Cry mutant mice reveals circadian tuning of photopic and mesopic retinal responses. J. Biol. Rhythms.

[15] Tso CF (2017). Astrocytes regulate daily rhythms in the suprachiasmatic nucleus and behavior. Curr. Biol..

[16] Brancaccio M, Patton AP, Chesham JE, Maywood ES, Hastings MH (2017). Astrocytes control circadian timekeeping in the suprachiasmatic nucleus via glutamatergic signaling. Neuron.

[17] Barca-Mayo O (2017). Astrocyte deletion of Bmal1 alters daily locomotor activity and cognitive functions via GABA signalling. Nat. Commun..

[18] Brancaccio M (2019). Cell-autonomous clock of astrocytes drives circadian behavior in mammals. Science.

[19] Newman EA (2004). A dialogue between glia and neurons in the retina: Modulation of neuronal excitability. Neuron Glia Biol..

[20] Newman, E. A. Glial cell regulation of neuronal activity and blood flow in the retina by release of gliotransmitters. *Philos. Trans. R. Soc. Lond. B Biol. Sci.* **370**, 20140195 (2015). 10.1098/rstb.2014.0195 PMC4455764 26009774

[21] Lehre KP, Davanger S, Danbolt NC (1997). Localization of the glutamate transporter protein GLAST in rat retina. Brain Res..

[22] Stone J, Dreher Z (1987). Relationship between astrocytes, ganglion cells and vasculature of the retina. J. Comp. Neurol..

[23] Huxlin KR, Sefton AJ, Furby JH (1992). The origin and development of retinal astrocytes in the mouse. J. Neurocytol..

[24] Reichenbach A, Bringmann A (2013). New functions of Müller cells. Glia.

[25] Bringmann A (2006). Müller cells in the healthy and diseased retina. Prog. Retin. Eye Res..

[26] Xu L (2016). Mammalian retinal Müller cells have circadian clock function. Mol. Vis..

[27] Mori T (2006). Inducible gene deletion in astroglia and radial glia—A valuable tool for functional and lineage analysis. Glia.

[28] Barca-Mayo O, Boender AJ, Armirotti A, De Pietri Tonelli D (2020). Deletion of astrocytic BMAL1 results in metabolic imbalance and shorter lifespan in mice. Glia.

[29] Smith CM (2019). The mouse Gene Expression Database (GXD): 2019 update. Nucleic Acids Res..

[30] Ekström P, Sanyal S, Narfström K, Chader GJ, van Veen T (1988). Accumulation of glial fibrillary acidic protein in Müller radial glia during retinal degeneration. Invest. Ophthalmol. Vis. Sci..

[31] Lananna BV (2018). Cell-autonomous regulation of astrocyte activation by the circadian clock protein BMAL1. Cell Rep..

[32] Rashid K, Akhtar-Schaefer I, Langmann T (2019). Microglia in retinal degeneration. Front. Immunol..

[33] Fan W (2022). Retinal microglia: Functions and diseases. Immunology.

[34] Riccitelli S, Di Paolo M, Ashley J, Bisti S, Di Marco S (2021). The timecourses of functional, morphological, and molecular changes triggered by light exposure in Sprague-Dawley rat retinas. Cells.

[35] Tosini G, Kasamatsu M, Sakamoto K (2007). Clock gene expression in the rat retina: Effects of lighting conditions and photoreceptor degeneration. Brain Res..

[36] Ripperger JA, Shearman LP, Reppert SM, Schibler U (2000). CLOCK, an essential pacemaker component, controls expression of the circadian transcription factor DBP. Genes Dev..

[37] Hiragaki S (2014). Melatonin signaling modulates clock genes expression in the mouse retina. PLoS ONE.

[38] Peirson SN (2006). Comparison of clock gene expression in SCN, retina, heart, and liver of mice. Biochem. Biophys. Res. Commun..

[39] Barca Mayo O, Berdondini L, De Pietri Tonelli D (2019). Astrocytes and circadian rhythms: An emerging astrocyte-neuron synergy in the timekeeping system. Methods Mol. Biol..

[40] Roska B, Werblin F (2001). Vertical interactions across ten parallel, stacked representations in the mammalian retina. Nature.

[41] Brunelli, R. *Template Matching Techniques in Computer Vision*. 10.1002/9780470744055 (Wiley, 2009).

[42] Baden T (2016). The functional diversity of retinal ganglion cells in the mouse. Nature.

[43] Tikidji-Hamburyan A (2015). Retinal output changes qualitatively with every change in ambient illuminance. Nat. Neurosci..

[44] Sagdullaev BT, McCall MA (2005). Stimulus size and intensity alter fundamental receptive-field properties of mouse retinal ganglion cells in vivo. Vis. Neurosci..

[45] Cameron MA, Barnard AR, Lucas RJ (2008). The electroretinogram as a method for studying circadian rhythms in the mammalian retina. J. Genet..

[46] Liu X, Zhang Z, Ribelayga CP (2012). Heterogeneous expression of the core circadian clock proteins among neuronal cell types in mouse retina. PLoS ONE.

[47] Felder-Schmittbuhl M-P (2018). Ocular clocks: Adapting mechanisms for eye functions and health. Invest. Ophthalmol. Vis. Sci..

[48] DeVera C, Baba K, Tosini G (2019). Retinal circadian clocks are major players in the modulation of retinal functions and photoreceptor viability. Yale J. Biol. Med..

[49] Baba K, Ribelayga CP, Michael Iuvone P, Tosini G (2018). The retinal circadian clock and photoreceptor viability. Adv. Exp. Med. Biol..

[50] Baba K (2018). Removal of clock gene Bmal1 from the retina affects retinal development and accelerates cone photoreceptor degeneration during aging. Proc. Natl. Acad. Sci. USA.

[51] Ait-Hmyed O (2013). Mice lacking Period 1 and Period 2 circadian clock genes exhibit blue cone photoreceptor defects. Eur. J. Neurosci..

[52] Ribelayga C, Cao Y, Mangel SC (2008). The circadian clock in the retina controls rod-cone coupling. Neuron.

[53] Zele AJ, Feigl B, Smith SS, Markwell EL (2011). The circadian response of intrinsically photosensitive retinal ganglion cells. PLoS ONE.

[54] Hong G (2018). A method for single-neuron chronic recording from the retina in awake mice. Science.

[55] Park SJ (2020). Connectomic analysis reveals an interneuron with an integral role in the retinal circuit for night vision. Life.

[56] Grimes WN (2021). A high-density narrow-field inhibitory retinal interneuron with direct coupling to Müller glia. J. Neurosci..

[57] Wang J (2017). Anatomy and spatial organization of Müller glia in mouse retina. J. Comp. Neurol..

[58] Schindelin J (2012). Fiji: An open-source platform for biological-image analysis. Nat. Methods.

[59] Liu X, Tang L, Liu Y (2017). Mouse müller cell isolation and culture. Bio Protoc..

[60] McCulloch DL (2015). ISCEV Standard for full-field clinical electroretinography (2015 update). Doc. Ophthalmol..

[61] Hilgen G (2017). Pan-retinal characterisation of light responses from Ganglion cells in the developing mouse retina. Sci. Rep..

[62] Maccione A (2014). Following the ontogeny of retinal waves: Pan-retinal recordings of population dynamics in the neonatal mouse. J. Physiol. (Lond).

[63] Umino Y, Solessio E, Barlow RB (2008). Speed, spatial, and temporal tuning of rod and cone vision in mouse. J. Neurosci..

[64] Dedek K (2008). Ganglion cell adaptability: Does the coupling of horizontal cells play a role?. PLoS ONE.

[65] Nathan J (2006). Scotopic and photopic visual thresholds and spatial and temporal discrimination evaluated by behavior of mice in a water maze. Photochem. Photobiol..

[66] Hilgen G (2017). Unsupervised spike sorting for large-scale, high-density multielectrode arrays. Cell Rep..

[67] Amin H, Nieus T, Lonardoni D, Maccione A, Berdondini L (2017). High-resolution bioelectrical imaging of Aβ-induced network dysfunction on CMOS-MEAs for neurotoxicity and rescue studies. Sci. Rep..

[68] Zeck GM, Masland RH (2007). Spike train signatures of retinal ganglion cell types. Eur. J. Neurosci..

[69] *Statistics and Machine Learning Toolbox—MATLAB*. https://www.mathworks.com/products/statistics.html.

