# Supplementary material for: Glial Bmal1 role in mammalian retina daily changes
Source: Sci Rep. 2022 Dec 13;12:21561. doi: 10.1038/s41598-022-25783-1 (PMC9747811; doi:10.1038/s41598-022-25783-1)
Supplement: Supplementary file 1 — Supplementary Figures. [file 41598_2022_25783_MOESM1_ESM.docx]

**Supplementary**

**
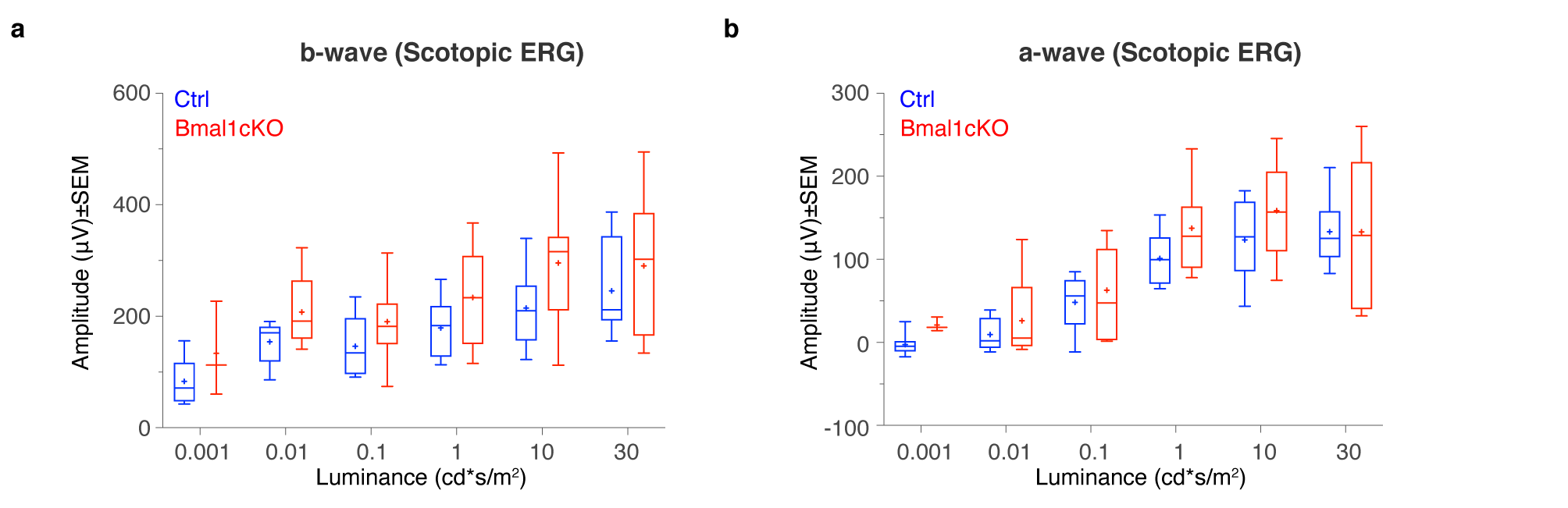
**

**Supplementary Fig. S1. Luminance-response functions for the b-wave (left) and a-wave (right) of the dark-adapted ERG responses recorded at ZT6. (a-b)** The quantification of fERG responses showed no significant differences between groups in b-wave (a) and a-wave (b) amplitudes under dark-adapted conditions (Scotopic ERG). The whiskers in the box plot represent the minimum and the maximum, “+” shows the mean. ERGs were performed at ZT6, n.s. vs. Ctrl, Two-way ANOVA. Ctrl (n=7, blue) and *Bmal1*cKO (n=7, red).


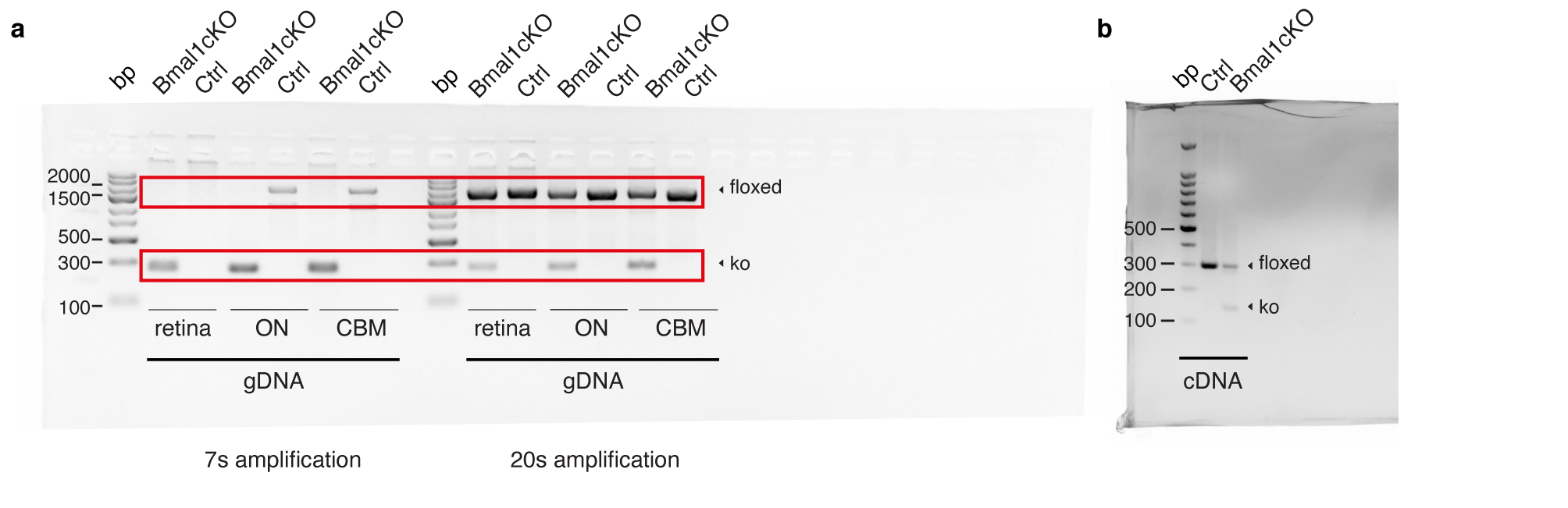


**Supplementary Fig. S2. Full gels from which the cropped images in Figure 1b and c of the main draft were cropped. (a)** PCR results show a band at ~270bp corresponding to the knockout allele in the retina, optic nerve (ON) and cerebellum (CBM) in *Bmal1*cKO. 7s of amplification time at 72°C in the PCR thermic profile is not enough to always amplify the larger floxed allele in both Ctrl and *Bmal1*cKO. **(b)** Amplified PCR products show fragments diagnostic of disrupted Bmal1 allele in cultured Müller cells of *Bmal1*cKO mice (PCR product 0.14 kb).


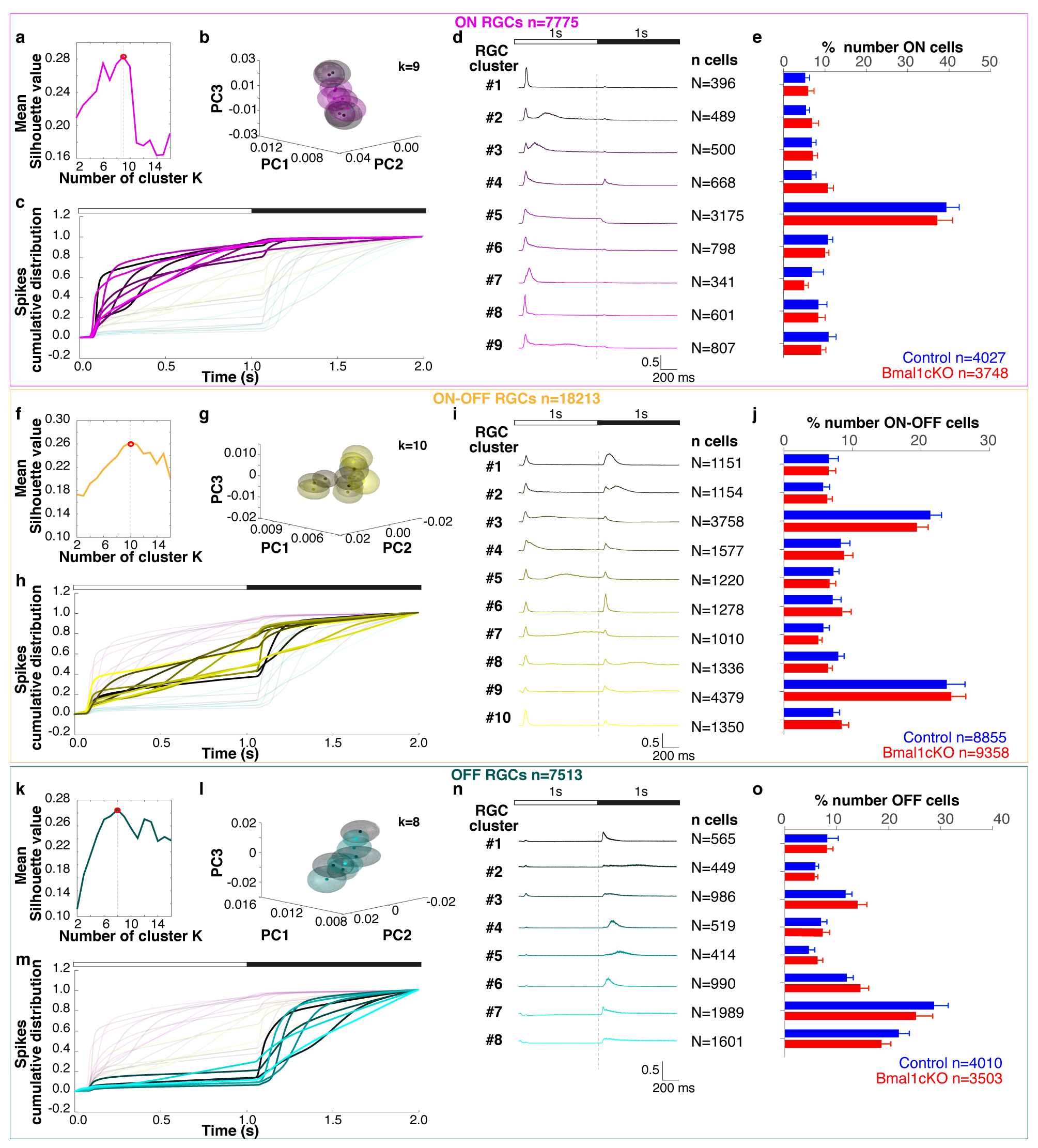


**Supplementary Fig. S3. Functional characterization of ON, ON-OFF, and OFF RGCs types found in Ctrl and *Bmal1*cKO retinae in photopic condition. (a-f-k)** The mean Silhouette value indicates the optimal number of clusters: 9 for ON RGCs, 10 for ON-OFF RGs, and 8 for OFF RGCs. The “k” represents the number of clusters that maximize the silhouette width. **(b-g-l)** Principal component analysis (PCA) of ON, ON-OFF, and OFF RGCs was carried out. **(c-h-m)** Cumulative spike trains distribution to alternating 1s white and 1s black flashes (20 repetitions, CT100) for ON, ON-OFF, and OFF RGCs. Each line represents the averaged response of the cells belonging to the color-coded cluster. **(d-i-n)** Population response of ON, ON-OFF, and OFF cells (9 ON, 10 ON-OFF, and 8 OFF clusters) per cluster. Each row shows the normalized PSTH of a single cluster. The numbers on the right of the PSTHs represent the numerosity of each cluster. **(e-j-o)** The histograms denote the relative proportion of recorded RGCs from each retina (Ctrl in blue and *Bmal1*cKO in red) assigned to the cluster. Data are shown as mean±SEM. Cluster numerosity does not exhibit bias toward one of the two strains (Multiple *t*-tests correct). On the bottom right of each panel, the total amount of ON, ON-OFF, and OFF RGCs recorded respectively in Ctrl and *Bmal1*cKO.


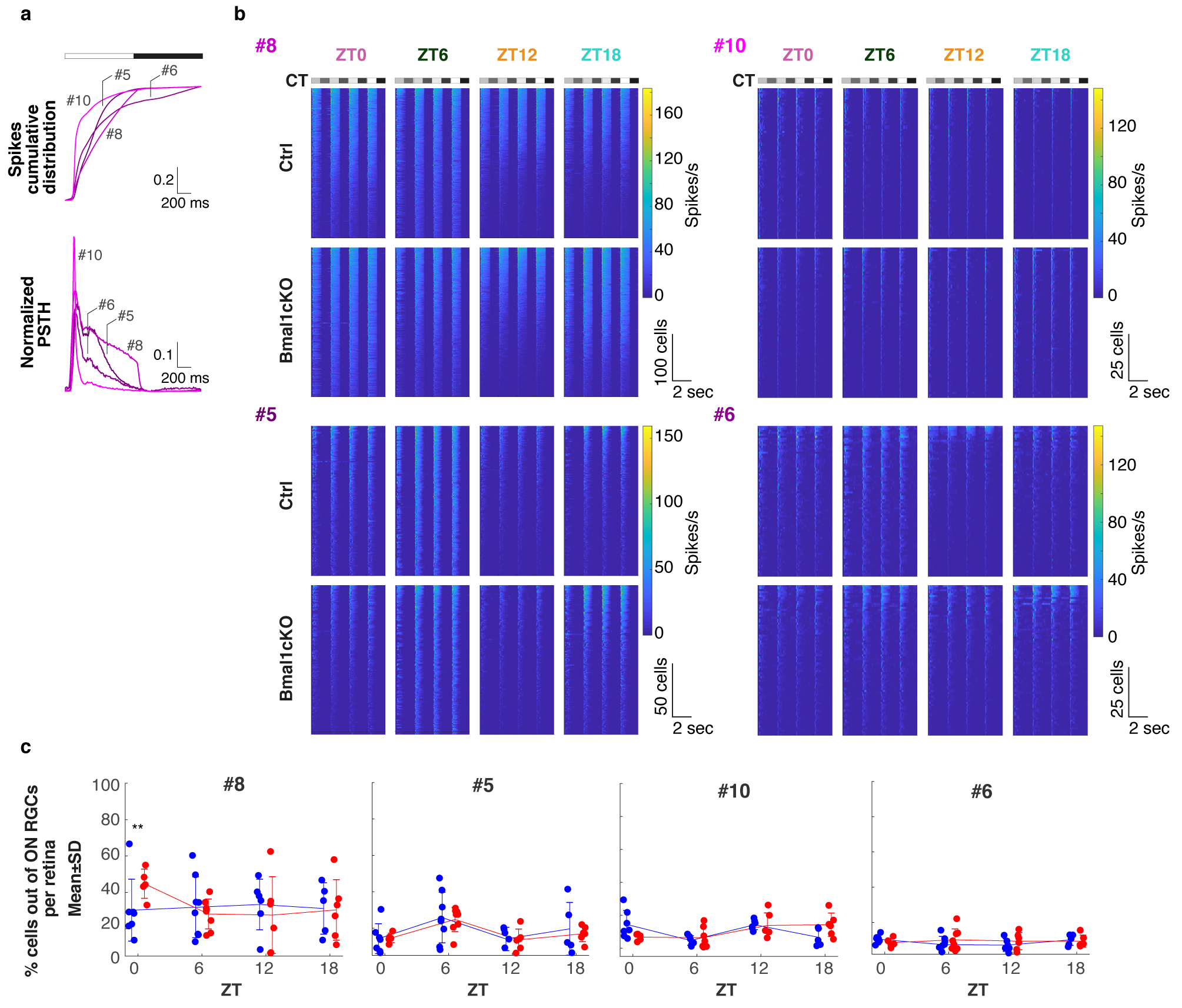


**Supplementary Fig. S4. Selected ON RGCs clusters overview. (a)** Average cumulative spike trains distribution (top) to alternating 1s white and 1s black flashes (stimulus indicated above) for selected ON RGCs clusters reported in Fig. 5 color-coded with the response averages (bottom panel from Fig. 5a). **(b)** Heat maps of concatenated light-evoked responses to the full-field stimuli at different contrasts (CTs 25-100, representative stimulus indicated above) of a subset of RGCs per cluster from different retinas sorted according to their mean firing rate. Each line represents the response of a single cell with activity color-coded such that yellow represent the highest activity. The number of plotted cells is determined by the minimum number of cells across ZTs and genotypes per cluster. **(c)** Percentage of cells per retina (single data points) allocated to each cluster out of the total ON RGCs recorded at different times of the day in Ctrl (blue) and *Bmal1*cKO (red). Data are shown as mean±SD. **P<0.01, Two-sample Kolmogorov-Smirnov test between groups at each ZT.


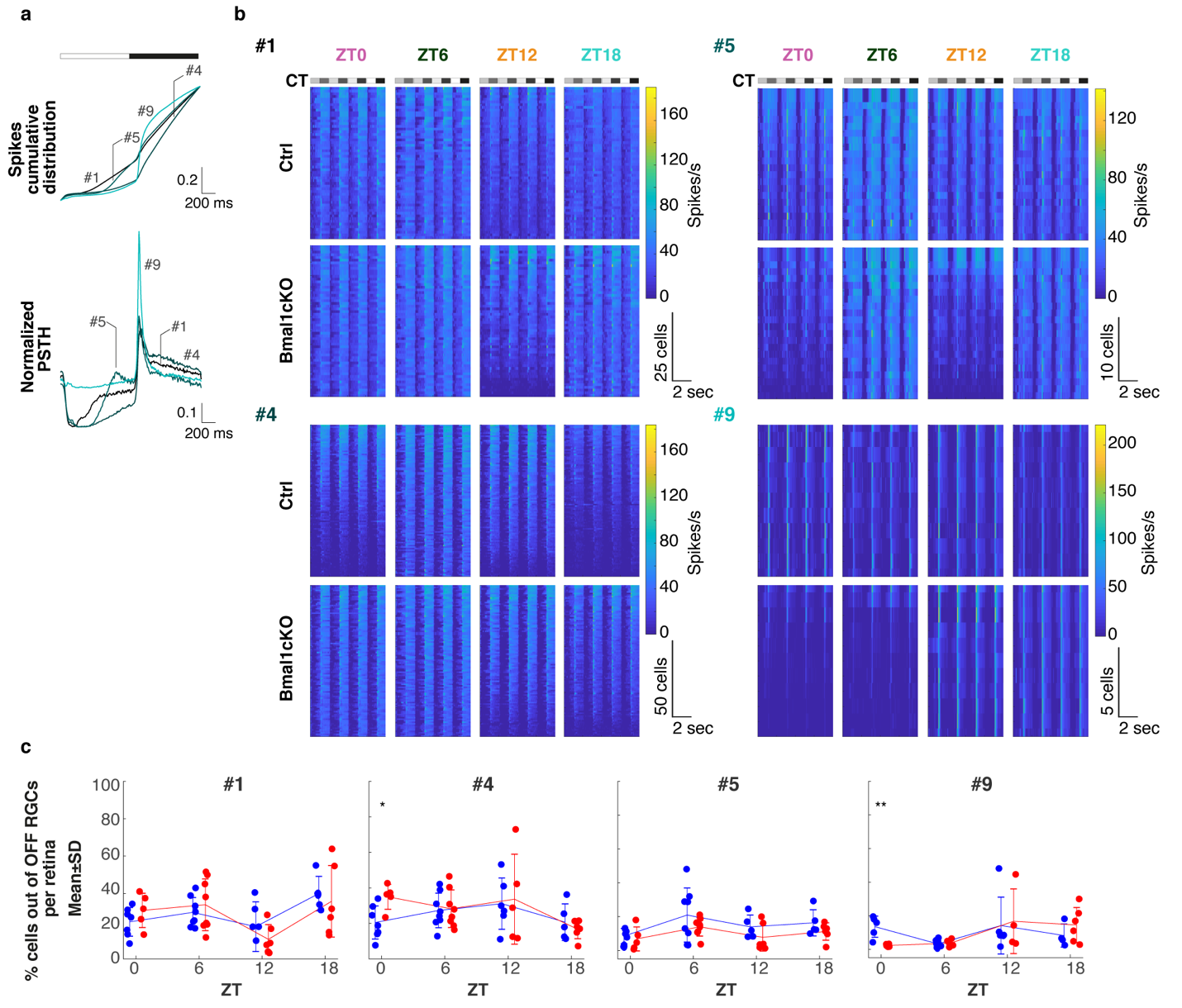


**Supplementary Fig. S5. Selected OFF RGCs clusters overview. (a)** Average cumulative spike trains distribution (top) to alternating 1s white and 1s black flashes (stimulus indicated above) for the selected OFF RGCs clusters in Fig. 6 color-coded with the response averages (bottom panel from Fig. 6a). **(b)** Heat maps of concatenated light-evoked responses to the full-field stimuli at different contrasts (CTs 25-100, representative stimulus indicated above) of a subset of RGCs per cluster from different retinas sorted according to their mean firing rate. Each line represents the response of a single cell with activity color-coded such that yellow represent the highest activity. The number of plotted cells is determined by the minimum number of cells across ZTs and genotypes per cluster. **(c)** Percentage of cells per retina (single data points) allocated to each cluster out of the total OFF RGCs recorded at different times of the day in Ctrl (blue) and *Bmal1*cKO (red). Data are shown as mean±SD. *P<0.05,**P<0.01, Two-sample Kolmogorov-Smirnov test between groups at each ZT.
